# Pancreatic cancer induces B cell lineage plasticity via Pax5 inhibition to sustain immunosuppression

**DOI:** 10.1038/s41420-026-03174-z

**Published:** 2026-06-02

**Authors:** Ali Kassem, Nataly Naser Al Deen, Sun Yifeng, Chau Fang, Laura Mayer, Lingling Zhang, Paul Kunath, Thomas Wirth, Uwe Knippschild, Mohammad Rahbari, Mathias Heikenwälder, Bo Kong, Cornelia Brunner, Alexander N. R. Weber, Nuh Rahbari, Hend Abdelrasoul

**Affiliations:** 1 Clinic for General and Visceral Surgery, University Hospital Ulm, Ulm, Germany; 2 Department of Medicine, Washington University in St. Louis, St. Louis, MO USA; 3 McDonnell Genome Institute, Washington University in St. Louis, St. Louis, MO USA; 4 Clinic for General, Visceral and Transplant Surgery, University Hospital Heidelberg, Heidelberg, Germany; 5 Department of Internal Medicine I, University Hospital of Ulm, Ulm, Germany; 6 Institute of Physiological Chemistry, University Ulm, Ulm, Germany; 7 Division of Chronic Inflammation and Cancer, German Cancer Research Center (DKFZ), Heidelberg, Germany; 8 The M3 Research Center, University Tuebingen, Faculty of Medicine, Institute for Interdisciplinary Research on Cancer Metabolism and Chronic Inflammation, M3-Research Center for Malignome, Metabolome and Microbiome, Otfried-Müller-Straße 37, Tübingen, Germany; 9 Cluster of Excellence iFIT (EXC 2180) “Image-Guided and Functionally Instructed Tumor Therapies, ” Eberhard-Karls University of Tübingen, Tübingen, Germany; 10 Department of Otorhinolaryngology and Head & Neck Surgery, Ulm University Medical Center, Ulm, Germany; 11 Ulm University Medical Faculty, Core Facility Immune Monitoring, Ulm, Germany; 12 Department of Innate Immunology, University hospital Tuebingen, Tübingen, Germany; 13 Clusters of Excellence EXC 2180 “Image Guided and Functionally Instructed Tumor Therapies” and EXC 2124 “Controlling Microbes to Fight Infections”, University of Tübingen, Tübingen, Germany; 14 Deutsches Konsortium für Translationale Krebsforschung (DKTK; German Cancer Consortium), Partner Site Tübingen, Tübingen, Germany; 15 Institute of Clinical and Experimental Trauma Immunology (ITI), University Hospital Ulm, Ulm, Germany

**Keywords:** Immunosurveillance, Immune evasion, B cells, Immunosurveillance

## Abstract

Pancreatic ductal adenocarcinoma (PDAC) is a highly aggressive tumor characterized by its ability to create an immunosuppressive tumor microenvironment. Here, using robust 3D co-culture systems, samples from PDAC patients and murine in vivo models, we described a novel immune evasion mechanism used by PDAC to inhibit the anti-tumor activity of B lymphocytes: We provide evidence that pancreatic cancer suppresses the B cell-specific transcriptional program while enforcing their reprogramming into functional macrophages. Thus, we hypothesize that B cells undergo transdifferentiation under the influence of PDAC, by losing their lymphoid identity and acquiring a myeloid immunosuppressive phenotype. This drastic change is enacted by the loss of *Pax5* expression. Importantly, our results showed that the Ex-B cells efficiently become phagocytic and produce soluble proteins that are known to enhance cancer cell survival and proliferation. This suggests that the PDAC-induced B cell to macrophage transdifferentiation pathway is functionally relevant and hence could serve as an immunotherapeutic target.

## Introduction

Pancreatic ductal adenocarcinoma (PDAC) is a highly aggressive tumor with an extremely poor survival rate, making it the third leading cause of cancer-related mortality [1]. PDAC is considered an immunologically “cold tumor” due to its low mutational burden and its profound immunosuppressive tumor microenvironment (TME). Although the TME of PDAC patients is enriched with immune cells [2], in most cases, they tend to be either dysfunctional effector cells or immunosuppressive cells such as FoxP3^+^ regulatory T cells, tumor-associated macrophages (TAMs), and myeloid-derived suppressor cells [3]. This immune landscape undermines the efficacy of immunotherapeutic approaches, leading to poor outcomes for PDAC patients [4]. The role of B cells in PDAC has only been appreciated over the last decade. B cells exert anti-tumor function through different mechanisms, including antibody-mediated cell cytotoxicity and activation of the complement cascade [5]. Importantly, the anti-tumor function of B cells mainly relies on their ability to differentiate into plasma cells producing antibodies against cancer targets [6]. In PDAC, B cells were found to occupy two distinct compartments: either sparsely infiltrated or organized in tertiary lymphoid structures (TLS), which are well-organized, non-encapsulated aggregates of immune cells and tumor stroma [7]. Effector B cells generated within TLS can further differentiate into antibody-secreting plasma cells or memory B cells. Additionally, the presence of B cells within TLS is associated with a “germinal center” immune signature, optimal infiltration and activation of T cells, improved response to immune checkpoint blockade therapy, and an overall favorable prognosis [8]. However, TLS formation is not a common feature of PDAC. In most cases, B cells are sparsely infiltrated in the TME, where they exhibit immune-regulatory functions. Regulatory B cells (B regs) suppress anti-tumor immunity by secreting anti-inflammatory cytokines and are associated with a poor prognosis and shorter overall survival [9]. However, the mechanisms regulating B cell recruitment or exclusion from PDAC TME and whether they exert pro- or anti-tumor function remain elusive. The induction of B regs is mediated by soluble proteins secreted in the TME, such as M-CSF, GM-CSF, and TSLP [10–12]. A similar strategy is employed by tumors to favor the generation and expansion of immune suppressive myeloid cells and TAMs, often at the expense of the B cell lineage. TSLP has been shown to induce early B cell mobilization from the bone marrow to the periphery in different cancer types [13, 14] and is also significantly elevated in patients with PDAC [12]. Beyond affecting the tumor immune landscape, we and others have previously reported that M-CSF can directly impact B cell lymphoid identity and induce their de-differentiation into macrophage-like cells under *Pax5* insufficiency [15, 16]. Importantly, both B cells and macrophages were shown to originate early, in the fetal liver, from bipotent B cell/macrophage progenitors [17, 18]. Additionally, oncogene-transformed B cell lines can undergo spontaneous and irreversible trans-differentiation to macrophages by the *Ras/Raf* oncogenes [19] or by the retroviral infection of the constitutively activated *Csf1r* in the presence of M-CSF in cell culture medium [20]. *Pax5* is a key player that regulates the commitment and maintenance of B-cell lymphoid identity. First, it collaborates with E2A, EBF1, and IKZF1 to guide the transition of uncommitted lymphoid progenitors into committed pro-B cells [21]. Subsequently, Pax5 activates transcription of B cell-specific genes such as *Cd19*, *IgH*, *VpreB*, and *Blnk*, while repressing non-B cell genes [15, 21], thus reinforcing B cell identity. This is exemplified in *Pax5*^*–/–*^ mice, which experience a complete block in B cell development at the pro-B cell stage in the bone marrow, resulting in a complete loss of mature B cells in the periphery [22]. Furthermore, *Pax5*^*–/–*^ pro-B cells demonstrate a broad multi-lineage potential, being able to de-differentiate into various hematopoietic cell types in the presence of appropriate cytokines as observed both in vitro and in vivo [16, 23–25]. Given the ability of several tumors to mobilize B cell precursors from the bone marrow into the periphery [21], together with the consistently elevated levels of M-CSF and GM-CSF in PDAC [26], we hypothesize that PDAC tends to “neutralize” B cells unless they exert regulatory functions, not only by inhibiting their anti-tumor activity but also by enforcing a rapid shutdown of the B cell-specific transcriptional program. Simultaneously, it compels their re-programming into functional macrophages, which we refer to as “Ex-B cells”. We also found that the Ex-B cells are phagocytic and produce soluble proteins that are known not only to enhance cancer cell survival and proliferation but also to maintain the immunosuppressive TME and attract more macrophages to the tumor site. Together, these findings suggest that PDAC-induced B cell reprogramming represents a functional immunoregulatory mechanism that might serve as a target for therapeutic intervention.

## Results

### 3D co-culture system mimicking PDAC TME induces loss of typical B cell features

**Fig. 1:**
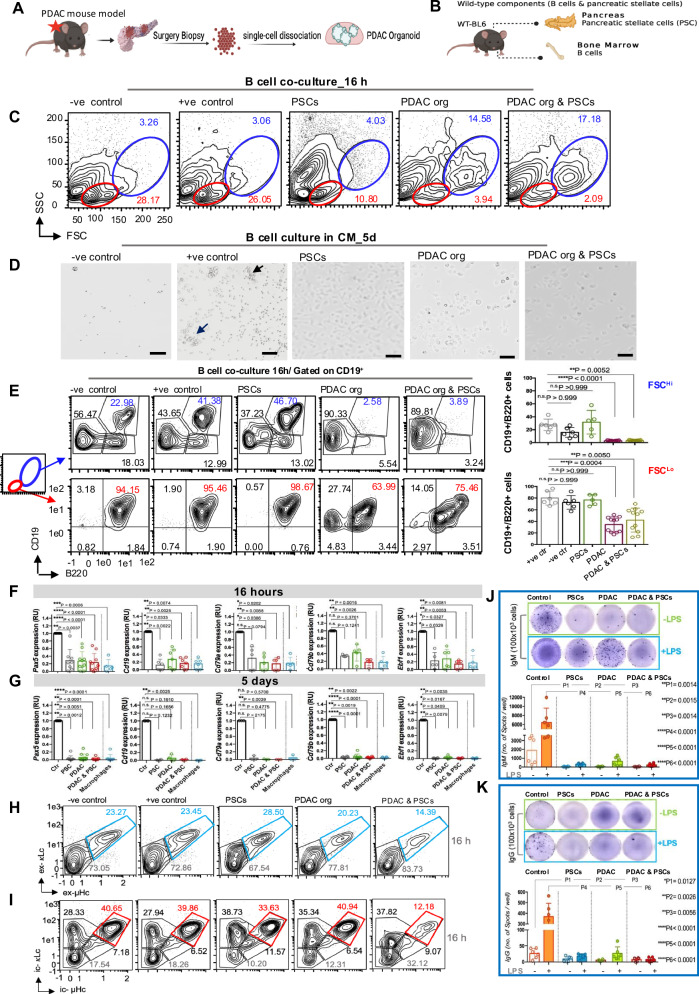
PDAC induces dramatic morphological and molecular changes in B lymphocytes. **A** Simplified illustration for the 3D culture system of pancreatic ductal adenocarcinoma cells (PDAC) isolated from two different mouse models. Acinar-derived *KPC*^*ERT2*^ model (*LSL-Kras*^*G12D+/–*^ *LSL; Trp53* ^*flox/flox*^*; P48 Cre*^*ERT2*^), Ductal-derived *SOX-KP*^*ERT2*^ model (*LSL-Kras*^*G12D+/–*^*; Sox9 Cre*^*ERT2*^*; R26RtdTomato*), **B** *Wildtype* (*wt*) cellular components used in the co-culture system. B cells were isolated from the bone marrow (BM) of C57BL/6 *wt* mice. Pancreatic stellate cells (PSCs) were isolated from normal pancreas of C57BL/6 *wt* mice. **C** Representative flow cytometry (FC) analysis of forward scattered (FSC) and sideward scattered (SSC) of B cells cultured in the two-way co-culture system under different conditions as indicated. Two different PDAC mouse organoid models were used in this experiment, but for simplification, results from the *KPC*^*ERT2*^ model are represented. B cells cultured in medium without cytokine supplements served as a negative survival control (-ve control), while cells cultured in medium supplemented with CM of the J558L cell line producing IL7 were used as a positive survival control (+ ve control). **D** Representative bright field microscopic pictures for a 5-day-old one-way culture of BM- derived B cells cultured under different conditions as indicated. 10×, size bar 50 μm. Black arrows refer to healthy B cell clusters that grow in suspension. Data are representative of at least 10 independent experiments. **E** Representative FC analysis showing the surface expression of the B lineage markers CD19 and B220 of BM- derived *wt* B cells cultured for 16 h in medium without cytokines (–ve survival control), medium supplemented with CM of J558L cells producing IL7 ( + ve survival control), PSCs, and PDAC mouse organoid with or without PSCs. Numbers in density plots indicate the percentages of cells in the respective gates. Data are representative of at least 5 independent experiments. Statistical significance of CD19^+^/B220^+^ cells in both large (FSC^Hi^/SSC^Hi^) (upper panel) and small (FSC^Lo^/SSC^Lo^) (lower panel) cell populations was calculated using the Kruskal-Wallis test and Dunn’s multiple comparisons test. *N* ≥ 6 per group, mean ± SD. Total RNA was isolated from B cells cultured under different conditions as indicated to detect the mRNA levels of the B lineage-specific genes: *Pax5, Cd19, Cd79a, Cd79b, and Ebf1*. Freshly isolated B cells, kept in PBS + 5% FBS, served as control (timepoint 0). Additionally, BM-derived cells cultured in medium supplemented with M-CSF for 5 days served as a control for conventional macrophage culture. Expression levels were measured by qRT-PCR after B cell co-culture for **F** 16 h or **G** 5 days. Data represent mean ± SD from at least 3 independent experiments and are shown as RU normalized control B cell culture. Statistical significance was calculated using the Kruskal-Wallis test and Dunn’s multiple comparisons test. FC plots illustrate the expression of B cell receptor (BCR) components: µ heavy chain (µHc) and kappa light chain (κLc) on BM-derived B cells cultured for 16 h under various conditions, as indicated. The detection of µHc and κLc was performed either **H** extracellularly (ex) or **I** intracellularly (ic). Numbers in the density plots indicate the percentage of BCR^+^ cells. **J** Plate readout of IgM ELISpot responses to different culture conditions for 3 days. A total 1 × 10^5^ purified B cells were stimulated with 2.5 µg/ml LPS (+ LPS) or left without stimulation (-LPS). B cells cultured in medium supplemented with 5% FBS served as a control. One-way ANOVA and Dunnett’s multiple comparisons test were used to compare IgM production under different culture conditions relative to the control. *N* = 6 measurements per group, mean ± SD. **K** Plate readout of IgG ELISpot responses to different culture conditions for 3 days. A total of 1 × 10^5^ purified B cells were stimulated with 2.5 µg/ml LPS (+ LPS) or left without stimulation (-LPS). B cells cultured in medium supplemented with 5% FBS served as a control. One-way ANOVA. Dunnett’s multiple comparisons test was performed to compare IgG production under different culture conditions relative to the control. *N* = 6 measurements per group, mean ± SD.

To investigate the impact of PDAC components on B cell developmental and functional decisions, we established 3D co-culture systems of B lymphocytes with the two major cell types that comprise the TME of PDAC: pancreatic cancer cells (PDAC organoids) and pancreatic stellate cells (PSCs), which serve as a source of cancer-associated fibroblasts in PDAC stroma [27]. Two mouse organoid models for PDAC were used: based on the acinar *KPC*^*ERTM*^ (*LSL-Kras*^*G12D+/–*^ *LSL; Trp53* ^*flx/fl*^*; P48 Cre*^*ERTM*^) [27], and the ductal *SOX9-KP*^*ERT2*^ (*LSL-Kras*^*G12D+/–*^*; Sox9 Cre*^*ERT2*^; *R26R*^*tdTomato*^) [28] (Fig. 1A), hereafter referred to as PDAC organoids. PSCs and B cells were isolated from the pancreas and bone marrow (bm) of *wildtype (wt) C57BL/6* mice, respectively (Fig. 1B). Both PDAC organoids and PSCs were seeded alone or in co-culture (Supplementary Fig. 1A). Finally, bm-purified CD19^+^/B220^+^ B cells were co-cultured with PDAC organoid, PSCs, or PDAC organoid & PSCs (Supplementary Fig. 1B). A “Two-way” co-culture system, allowing mutual exchange of the cytokines and soluble components, was used for the short-term B cell co-culture (16 h). For long-term culture (5- 7 d), a “one-way” culture system in which conditional media (CM) of PSCs, PDAC, or PDAC & PSCs cultures were collected, sterile filtered, and used to culture B cells (Supplementary Fig. 1C) [29]. Indeed, both PDAC models caused striking, rapid, and progressive morphological changes in B cells. Compared to controls, B cells co-cultured either with PDAC organoid alone or PDAC organoid & PSCs demonstrated significant heterogeneity in terms of cell size and granularity. Based on forward (FSC) and side scatter (SSC), the small FSC^Low^/SSC^Low^ population, typically assigned as B cells, decreased by a factor of 28 to only 2%, whereas large FSC^high^/SSC^high^ cells increased from 3 to 17% in the presence of PDAC organoid & PSCs (Fig. 1C). Remarkably, this alteration occurred within just 16 h of co-culture. Furthermore, the adherence behavior of B cells cultured for 5 days in the CM of PDAC components changed from suspension growth to adherence (Fig. 1D). Collectively, we thus note a profound change in B cell morphology as a consequence of exposure to PDAC CM or organoids.

### B cells co-cultured under PDAC conditions lose surface markers and transcriptional B lineage identity

Since we observed clear alterations in B cell morphology, we verified B cell identity by checking the surface expression of CD19 and B220 on the small and large cells after 16 h of co-culture. Indeed, FSC^high^/SSC^high^ cells downregulated CD19 and completely lost B220 expression (Fig. 1E, upper panel), while in FSC^Low^/SSC^Low^ cells, downregulation was more moderate (Fig. 1E, lower panel), indicating a critical disturbance in B cell identity. As Pax5 is also classically known as the “guardian” of B cell identity [30], we investigated *Pax5* expression levels over the course of these changes. Intriguingly, *Pax5* expression levels decreased significantly and rapidly in samples exposed to PDAC components, and this coincided with decreased expression of dependent B cell-specific genes such as *Cd19, Cd79a* (coding for CD79a/Igα), *Cd79b* (CD79b/Igβ), and *Ebf1*. The reduction of *Pax5* and its related genes was initially observed after 16 h of the co-culture (Fig. 1F) and progressed to a complete loss of expression after 5 d (Fig. 1G). This resembles the expression levels in bm macrophages, analyzed as a control (Supplementary Fig. 1D). These findings highlight the crucial role of *Pax5* in maintaining B lymphoid lineage commitment in the context of pancreatic cancer and the consequences of its loss upon PDAC exposure.

### PDAC enforces rapid downregulation of BCR and renders B cells non-functional

Next, we sought to determine the extent to which the PDAC-induced changes affected B cell functionality, e.g., B cell receptor (BCR)-mediated B cell activation and plasma cell differentiation [31], which have been described in several tumors and, in most cases, are linked to a favorable prognosis [32]. Our data showed that B cells co-cultured with PDAC & PSCs were unable to efficiently express the BCR on the cell surface, as evidenced by a reduced expression of κ light and µ heavy chains (Fig. 1H). Since BCR internalization and Igα/Igβ downregulation could be indicators for B cell activation, we also checked the intracellular expression of BCR components since RNA levels of Igα (*Cd79a*) and Igβ (*Cd79b*) indeed had decreased (*cf*. Fig. 1F, G). Interestingly, we found that B cells co-cultured with PDAC & PSCs had lower intracellular BCR expression compared to B cells cultured under other conditions (Fig. 1I). Next, it was crucial to evaluate the functional consequences of BCR downregulation by examining the antibody secretion capacity of the B cells cultured under PDAC conditions. Indeed, B cells exposed to PDAC components showed markedly reduced IgM and IgG secretion compared to control (Fig. 1J, K). Upon the LPS stimulation, IgM and IgG secretion could be slightly restored in B cells cultured either with PSCs or PDAC alone. However, the combination of PDAC organoids and PSCs exerted the most profound inhibitory effect regardless of the LPS stimulation. This is consistent with the observed reduction in BCR expression.

### PDAC activates the transcription of non-lymphoid genes

**Fig. 2:**
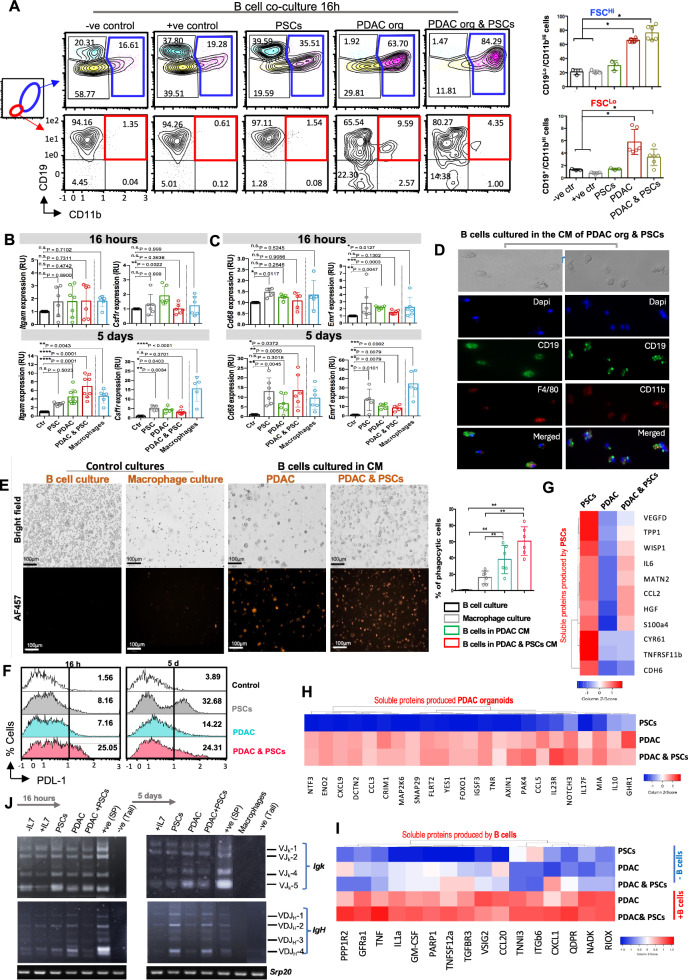
B lymphocytes acquire a myeloid phenotype in PDAC TME. **A** Representative FC plots showing the surface expression of the B lineage marker CD19 and the myeloid marker CD11b of BM-derived B cells cultured for 16 h in different conditions as specified. Numbers in density plots indicate the percentages of cells in the respective gates. B cells cultured in medium without cytokine supplements served as -ve control, while cells cultured in medium supplemented with CM of the J558L cell line producing IL7 were used as +ve survival control. Statistical analysis of CD19^Lo^CD11b^+^ cells in both large (FSC^Hi^) (upper panel) and small (FSC^Lo^) (lower panel) cell populations. Data are representative of at least 3 independent experiments. Statistical significance was calculated using the Kruskal-Wallis test and Dunn’s multiple comparisons test. *N* ≥ 3 per group, mean ± SD. **B** Transcript levels of the myeloid lineage markers, *Itgam* (CD11b gene), and *Csf1r* were measured after 16 h (upper panel) or 5 d (lower panel) of the co-culture. Statistical significance was calculated using the Kruskal- Wallis test, Dunn’s multiple comparisons test. *N* ≥ 6 per group, mean ± SD. **C** Macrophage-specific genes *Cd68 and Emr1* (the gene encodes F4/80) were assessed by qRT-PCR after B cell co-culture for 16 h (upper panel) or 5 d (lower panel) in different conditions as indicated. Statistical significance was calculated using the Kruskal-Wallis test and Dunn’s multiple comparisons test. *N* ≥ 5 per group, mean ± SD. For B & C, Freshly isolated B cells, kept in PBS + 5% FBS, served as control (timepoint 0). Additionally, BM-derived cells cultured in medium supplemented with M-CSF for 5 days served as a control for conventional macrophage culture. **D** Brightfield and fluorescence immunocytochemistry of B cells cultured for 5 days in the CM of PDAC organoid & PSCs. Anti-CD19, anti-CD11b, and anti-F4/80 antibodies were incubated at 1:200 dilution (blue, Dapi; green, CD19; red (left): F4/80; red (right): CD11b. Scale bar = 15 μm. **E** Representative Brightfield and fluorescence immunocytochemistry of a phagocytosis assay for B cells cultured in the CM of PDAC organoid or PDAC organoid & PSCs for 5 d. The phagocytic capacity was tested by adding Zymosan labeled for fluorescence at Ex/Em 540/570 nm (ab234054, red Zymosan). Statistical analysis illustrates the percentage of phagocytic cells after a 3 h exposure to red Zymosan fluorescent beads. Conventional B cell and macrophage culture involved as controls for the experiment. Statistical significance was calculated using the unpaired student T- test, Dunn’s multiple comparisons test. *N* = 6 per group, mean ± SD. **F** FC analysis of the PDL-1 expression of B cells cultured under PDAC conditions compared to control for 16 h (left) or 5 d (right). Numbers indicate signal intensity of PDL-1. Heat map showing main soluble proteins produced by **G** PSCs, **H** PDAC organoid, or **I** B cells after their exposure to PDAC components. Basal ISCOV´s media were used as a control to subtract the background secretion. Colors are assigned according to the relative scale of expression and represent a fold-increase change versus the co-culture condition. **J** Semi-quantitative PCR of the *Igk* rearrangements of the *V*_*k*_ gene segments to *J*_*κ*_*1, J*_*κ*_*2, J*_*κ*_*4, and J*_*κ*_*5* gene segments (upper right). *IgH* rearrangements of the proximal *VD*_*h*_ gene segments to *J*_*H*_*1, J*_*H*_*2, J*_*H*_*3, and J*_*H*_*4* gene segments (lower right). The gDNA input was normalized to *Srp20* PCR products. Genomic DNA (gDNA) was isolated from B cells cultured under PDAC conditions as specified after 16 h or 5 d of culture. GDNA isolated from splenic B cells was utilized as a +ve control for *V(D)J* recombination assay, while gDNA isolated from the mouse tail served as a technical –ve control for the recombination analysis. For comparison, a culture of conventional macrophages was generated concurrently with the B cell treatment. B cells cultured either in medium supplemented with IL7 (+ IL7) or without IL7 (-IL7) were used as +ve and -ve controls for survival, respectively.

Besides inducing B lymphoid-specific genes, Pax5 restricts the B lineage-inappropriate transcription programs [33]. The profound reduction in *Pax5* expression level and its related B cell-specific genes, along with the alterations in their morphology and adhesion behavior in B cells subjected to PDAC conditions, were all indicative of a shift potentially towards a myeloid phenotype. Therefore, we examined the expression of myeloid markers known to be repressed by Pax5, such as *Itgam* (coding for CD11b) and *Csf1r* [33]. Notably, we found that the downregulation of *Pax5* coincided with the expression of both *Itgam* and *Csf1r* (Fig. 2A, B), reminiscent of their high expression observed in conventional macrophage culture, analyzed as a control for myeloid gene expression. These findings further confirm that PDAC can manipulate B cells by eliminating B cell identity, on the levels of morphology, surface markers, gene and BCR expression, and antibody secretion. Collectively, this begged the question of which new phenotypes these viable cells acquired as a result.

### PDAC induces transdifferentiation of B cells to functional macrophages with immunosuppressive characteristics

To characterize the identity of the “Ex-B cells”, the observed similarity to macrophage-expressed genes (*cf*. Fig. 2A, B) prompted us to consider transdifferentiation of B cells to macrophages. Notably, studies reporting instability of B cell identity predominantly utilized *Pax5*-deficient, and hence unstable, pro-B cells with non-rearranged BCR, whereas our B cells are *Pax5*-*wt*. Nevertheless, when we investigated the macrophage-specific markers CD68 and F4/80, the Ex-B cells showed expression levels comparable to bona fide macrophages. (Fig. 2C, D, and Supplementary Fig. 2A–F). Moreover, these cells showed an acquired capacity to phagocytose fluorochrome-labeled zymosan particles. Notably, while B cells cultured under standard B cell conditions did not exhibit any phagocytic activity, Ex-B cells grown under PDAC conditions showed enhanced phagocytic capacity. Ex-B cells grown in the CM of PDAC & PSCs exceeded in their phagocytosis capacity cells grown in the CM of PDAC alone, and both engulfed the fluorescent beads more efficiently than conventional macrophages (Fig. 2E). As tumor-associated macrophages are often immunosuppressive [34, 35], it was then important to investigate whether these cells exhibited immunosuppressive characteristics. To this end, we analyzed the expression of PDL-1 on the Ex-B cells. Our analysis revealed a clear upregulation of PDL-1 expression on B cells exposed to PDAC components (Fig. 2F). Accordingly, we evaluated the direct impact of Ex-B cells on CD8^+^ T cell activation and proliferation. For that, purified CD8^+^ T cells were either activated using CD3/CD28 beads or left unstimulated. T cells were then co-cultured with Ex-B cells at increasing Ex-B: T ratios (1:1, 2:1, and 4:1). We observed that activated CD8^+^ T cells cultured with Ex-B cells showed a reduction in Ki-67 expression compared to control T cells cultured without Ex-B cells. Notably, this inhibitory effect was dose- dependent and became more pronounced with increasing Ex-B: T cell ratios (Supplementary Fig. 3A). Additionally, we also assessed the activation status of CD8^+^ T cells by measuring CD69 expression. As expected, while CD3/CD28 stimulation induced a robust upregulation of CD69, direct co-culture with Ex-B cells resulted in a reduction in CD69 expression, regardless of the activation status of CD8^+^ T cells (Supplementary Fig. 3B). Together, these findings demonstrate that Ex-B cells exert a direct immunosuppressive effect on CD8^+^ T cells by impairing both activation and proliferation capacity.

We then analyzed the cellular source of the soluble proteins produced in the CM of 48-h PSCs, PDAC, or PDAC & PSCs co-cultures, as well as CM from 24-h one-way B cell cultures (Supplementary Fig. 3C). We classified the soluble proteins into three categories: (A) produced by PSCs (Fig. 2G), (B) produced by PDAC (Fig. 2H), and (C) produced by Ex-B cells (Fig. 2I). Olink secretome analysis showed that PSCs were responsible for the production of the pro-inflammatory cytokines: IL-6, CCL2, and HGF, which are known to induce macrophage differentiation to M2 phenotype and thereby influence immune regulation of adaptive immune cells [36–39]. Additionally, CCL2 can recruit tumor necrosis factor (TNF)-α-secreting macrophages, which in turn induce the reprogramming of classical PDAC cells into a more aggressive phenotype [40]. Furthermore, the CM from PDAC and PDAC & PSCs cultures was enriched with IL-10, suggesting a potential role in inducing an immunosuppressive macrophage phenotype. Meanwhile, the elevated levels of pro-inflammatory factors such as IL-17, CCL5, and CXCL9 may contribute to the recruitment of regulatory immune cells, shaping the overall immunosuppressive landscape of PDAC [41]. Moreover, compared to the secretome of co-cultures lacking B cells, proteins typically secreted by M2 macrophages in response to inflammatory stimuli were highly increased, e.g., TNF, CCL20, IL-1α, and CXCL1 [41–43]. We have also observed increased levels of PARP1, Poly (ADP-ribose) polymerase 1, a factor known to induce lineage commitment towards macrophages [44]. Finally, our data demonstrated that the CM of PDAC& PSCs contains elevated levels of M-CSF and GM-CSF compared to control medium (Supplementary Fig. 3D). Surprisingly, we also found that Ex-B cells themselves produce GM-CSF, suggesting a positive feedback loop to maintain their acquired myeloid nature. This finding suggests that the PDAC TME provides sufficient M-CSF to support the acquisition of macrophage phenotype by Ex- B cells.

### Ex-B cells are of confirmed B lineage origin

To exclude the possibility that the Ex-B cells originated from macrophage contaminants in the B cell culture, we analyzed heavy (*IgH)* and light (*IgL)* chain recombination patterns, a unique genetic “fingerprint” of B cells, in the growing cells. Whereas these are not rearranged in the macrophage lineage, B cells first rearrange *V(D)J* segments in *IgH*, and subsequently VJ segments in *IgL* (*Igk* or *Igλ)* [45, 46]. Thus, only macrophages with a B cell origin would be expected to have re-arranged *IgH* and *IgL* loci. To test this, we utilized semi-quantitative PCR with primers as previously described [47]. Different controls were utilized alongside the experimental samples to ensure the robustness of the experimental design. For example, freshly purified B cells acted as a timepoint zero control (B cells without culture incubation). B cells cultured in a cell culture medium supplemented with interleukin 7 ( + IL7) served as a positive control for B cell survival, while those cultured in a basic medium devoid of cytokines (-IL7) served as a negative control for B cell survival. Splenic B cells were utilized as a positive technical control for *V(D)J* recombination, while genomic DNA extracted from mouse tail tissue served as a negative technical control. Additionally, genomic DNA isolated from a 5-day-old conventional macrophage culture was included as an extra experimental control for cells that do not undergo *V(D)J* recombination. Indeed, both the control B cells (cultured in -IL7, +IL7, or timepoint zero) and the Ex-B cells showed comparable *V*_*k*_*J*_*K*_ and *V*_*H*_*DJ*_*H*_ recombination patterns for the *IgK* **(**Fig. 2J and Supplementary Fig. 3E, left panel) and *IgH* genes (Supplementary Fig. 3E, right panel), respectively. Notably, the growing Ex-B cells collected for the *V(D)J* recombination analysis on day 5 were all adherent, indicating a complete reprogramming of the cultured B cells. To further validate the B cell origin of Ex-B cells, we performed FACS-based sorting of CD19^+^B220^+^ B cells from the bone marrow of the Pan-RFP reporter mouse line (*Ac RFP*) [48] (Supplementary Fig. 4A–C). The purified *RFP*^+^ B cells were then cultured either under standard B cell conditions or in the CM of PDAC & PSCs. Consistent with our previous observation, CM induced the trans-differentiation of the *RFP*^+^ B cells into adherent cells (Supplementary Fig. 4D). We also noticed that the reprogrammed Ex-B cells displayed reduced RFP signal intensity compared to the original B cell population. This reduction in reporter signal during lineage reprogramming has been described previously and is thought to reflect transcriptional or metabolic changes associated with lineage reprogramming [49]. Although RFP expression in the system used is not specific to B cells, the sorting strategy yielded a highly purified population of B cells as verified by CD19 and B220 surface markers expression (*cf*. Supplementary Fig. 4B, C). Finally, the identification of the B cell-specific fingerprint, together with the transdifferentiation of the *RFP*^+^ B cells, allows us to exclude the possibility of myeloid contamination and confirms that Ex-B cells originate from bona fide B cells.

### Ectopic expression of *Pax5* prevents B cell re-programming but is insufficient to sustain B cell survival in PDAC TME

To determine whether the stabilization of Pax5 expression can rescue B cell identity in PDAC, CD19^+^B220^+^ B cells were retrovirally transduced with *Pax5-*IRES*-*GFP expression vector and subsequently cultured either under PDAC conditions or under standard B cell culture conditions (Supplementary Fig. 5A). Pax5 expression resulted in the stabilization of CD19 expression under PDAC conditions, hence, could rescue the lymphoid identity of B cells. However, the frequency of the *Pax5*-GFP^+^ B cells markedly declined from ~21% at day 1 to ~4% at day 5 when cultured under PDAC conditions. In contrast, *Pax5*-GFP^+^ B cells cultured under the standard B cell conditions increased from ~21% at day 1 to ~24% at day 5, indicating a stable maintenance of *Pax5*-expressing cells. (Supplementary Fig. 5B–D). This suggests that while enforced *Pax5* expression is sufficient to maintain B cell identity, as evidenced by the high CD19 expression, it could not sustain B cell viability in PDAC TME. Together, this data suggests that PDAC either drives B cells to acquire a myeloid phenotype, supporting its immunosuppressive nature or induces the loss of resistant clones that might express high *Pax5* levels.

### M2-like tumor-associated macrophages express B-cell-specific markers

**Fig. 3:**
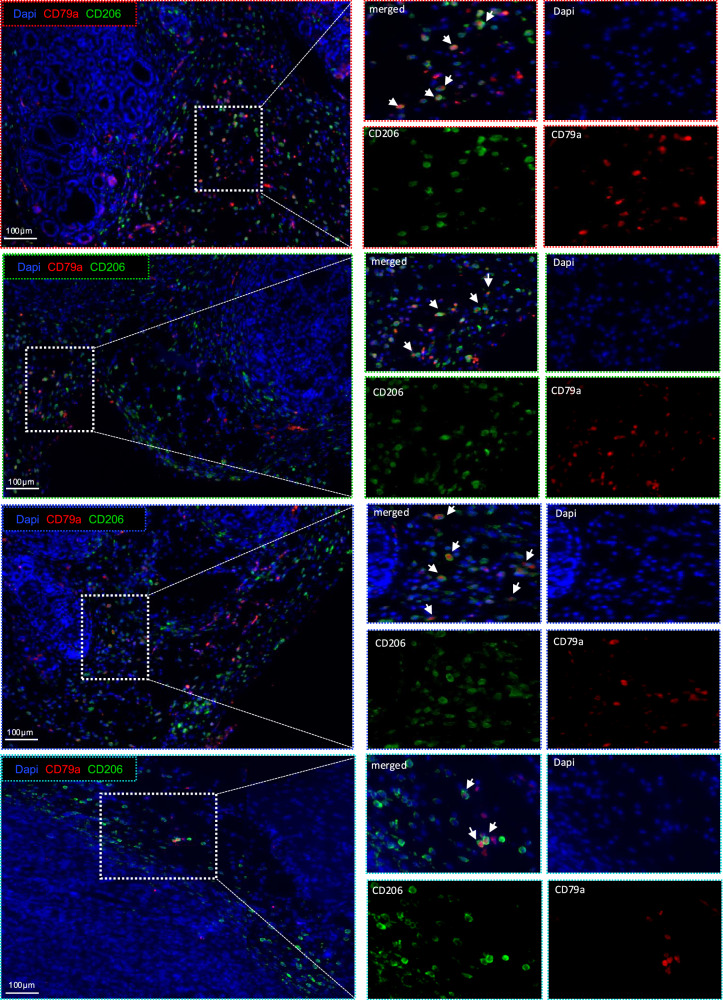
Tumor-associated macrophages express the B cell-specific marker CD79a. Representative immunofluorescence (IF) staining of PDAC from Two different PDAC mouse models: the cerulein- injected *KC* model (*LSL-Kras*^*G12D+/–*^; *Pdx1-Cre)* (upper three panels) and the *KPC* model *(LSL-Kras* ^*G12D+/–*^ *LSL; Trp53* ^*fl/fl*^*; Pdx-1 Cre)* (lower panel), and The staining shows the CD206^+^ M2 tumor- associated macrophages (green) express the B cells- specific marker CD79a (red).

**Fig. 4:**
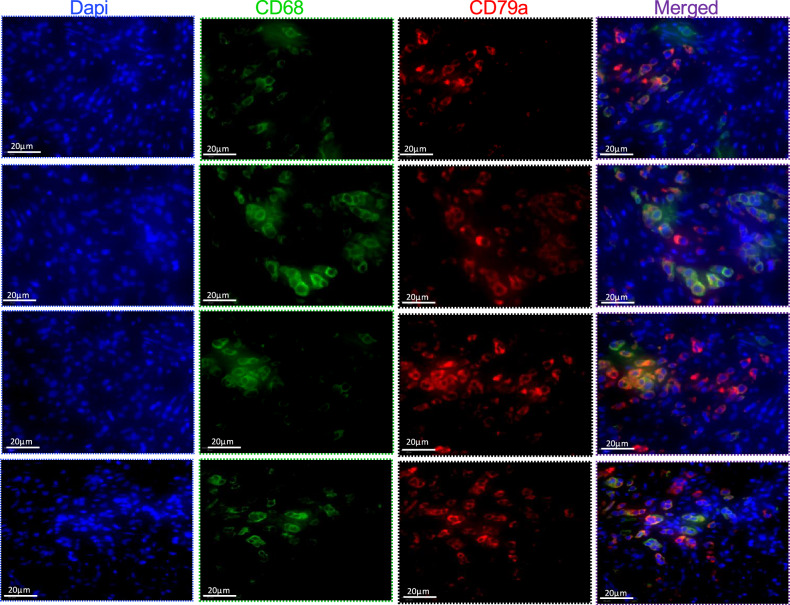
CD68^+^ TAMs express CD79a in PDAC patients. Representative IF staining of formalin- fixed paraffin- embedded PDAC tissue sections from different patients. The staining shows CD68^+^ tumor-associated macrophages (green) co-express the B cell-specific marker CD79a (red). Scale bars: 20 µm.

It was important to investigate whether our in vitro observation can be validated on the in vivo level by investigating whether the TAMs in PDAC express B-cell-specific markers. For that, we performed multiplex immunofluorescence staining for paraffin-embedded tumor tissues from two different PDAC mouse models, including *KPC (LSL-Kras*^*G12D+/–*^*; Trp53*^*fl/fl*^*; Pdx1-Cre)* [50] and cerulein-injected KC (*LSL-Kras*^*G12D+/–*^; *Pdx1-Cre)* [51]. Importantly, we detected that the CD206^+^ M2- like TAMs express the B cell-specific marker CD79a (Fig. 3). Interestingly, we further confirmed the presence of CD68^+^ TAMs that express the B cell-specific marker CD79a in PDAC patients (Fig. 4). It is noteworthy to mention that, while Ex-B cells were detected as aggregated infiltrates within the tumor tissue, B cells in larger accumulations seemed to be protected from this inhibitory effect (Supplementary Fig. 6). This observation further supports our hypothesis that PDAC-induced B cell transdifferentiation is a mechanism which is used by PDAC to escape immune monitoring.

### PDAC disturbs normal hematopoiesis in the bone marrow of tumor-bearing mice

**Fig. 5:**
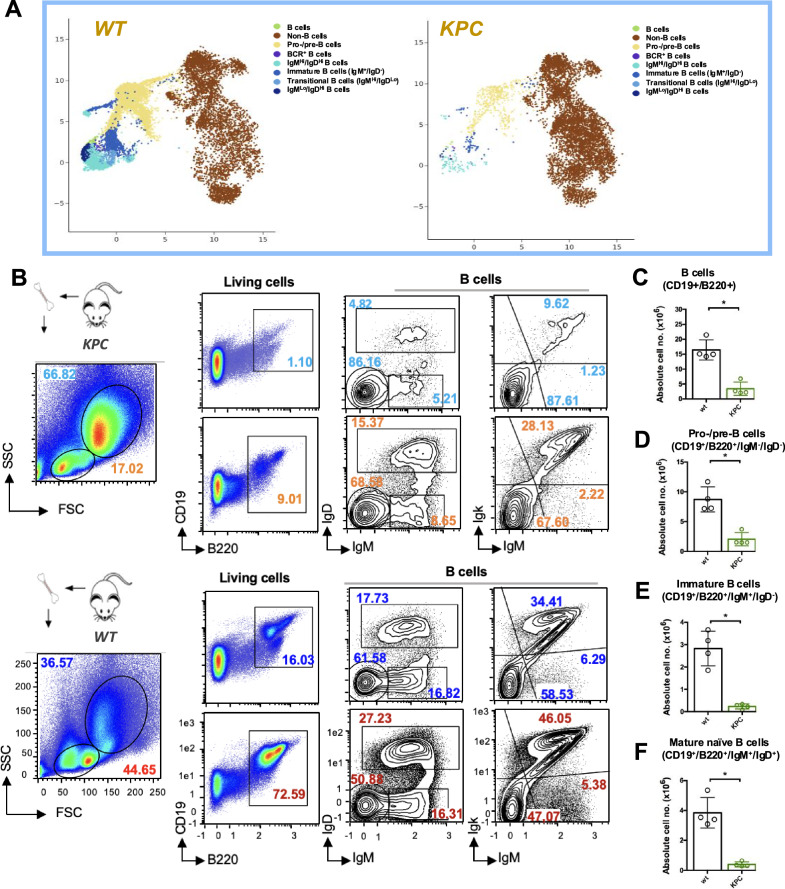
Altered immune cell homeostasis and defective B cell development in the bone marrow of the *KPC*^*ERT2*^ mouse model. **A** T-Distributed Stochastic Neighbor Embedding (T-SNE) plot shows the distribution of different B cell subsets versus non-B cell compartments in the BM of 8–12 weeks-old *KPC* model (*LSL-Kras* ^*G12D+/–*^ *LSL; Trp53* ^*fl/fl*^*; Pdx1 Cre)* and age-sex matched *wildtype* control. T-SNE plots were generated based on the CD19, B220, IgM, IgD, and IgK expression used for the FC analysis. T-SNE plots were prepared by the Cytolution Platform v1.1.0 provided by Cytolitics. **B** Representative FC analysis of bone marrow-derived cells from the PDAC mouse model; *KPC*^*ERT2*^ (upper) *and age-gender matched wt control* (lower). *The* analysis from left to right shows: FSC/ SSC: lymphoid population (FSC^Lo^/SSC^Lo^) and myeloid population (FSC^Hi^/SSC^Hi^). Fixable viability dye was used to label dead cells. CD19/B220 expression (pre-gated on living cells): B cells (CD19^+^/B220^+^) or non-B cells (CD19^-^/B220^-^). Representative plots for expression of the BCR isotypes (IgM/IgD) showing different B cell developmental stages (pre-gated on CD19^+^/B220^+^): Pro-/pre-B cells (IgM^-^/IgD^-^), Immature B cells (IgM^+^/IgD^-^), and mature B cells (IgM^+^/IgD^+^). FC plots show BCR expression (Igk/IgM) pre-gated on B cells (CD19^+^/B220^+^). Representative data of 4 mice per genotype are shown. Numbers in FC plots indicate the percentages of the cells in the respective gates. Quantification of the absolute cell numbers of **C** Total B cells, **D** Pro-/pre-B cells, **E** Immature B cells, and **F** Mature B cells in BM from control (*n* = 4), and *KPC*^*ERT2*^ model (*n* = 4). Circles indicate data from individual mice. Statistical significance was calculated by applying the unpaired t-test.

Recent studies have shown that the progression of certain solid tumors is associated with profound perturbations in lymphoid hematopoiesis and a skewing toward myeloid cell lineages [13]. We questioned whether PDAC can also impact normal B cell lymphopoiesis and development in the bone marrow of the PDAC-bearing mice. To address that, we conducted immunophenotypic analysis of bone marrow-derived cells from *KPC* (*LSL-Kras*^*G12D+/–*^*; Trp53*^*fl/fl*^*; Pdx1-Cre)* mice and age- and sex-matched *wt C57BL/6* controls. Our analysis revealed that the bone marrow of the *KPC* is heavily occupied by non-B cells (Fig. 5A), while the B cell population (CD19^+^B220^+^) is significantly diminished in *KPC* compared to control mice (Fig. 5B, C). We also observed a marked reduction in the absolute cell numbers of the different B cell subsets, including pro-/pre-B cells (CD19^+^B220^+^gM^-^IgD^-^) (Fig. 5D), immature B cells (CD19^+^B220^+^IgM^+^IgD^-^) (Fig. 5E), and mature naïve B cells (CD19^+^B220^+^IgM^+^IgD^+^) (Fig. 5F). Importantly, we also detected significantly fewer cells expressing the BCR (IgM^+^IgK^+^ cells) in the *KPC* mice compared with their *wt* littermates. This is consistent with our in vitro results showing that PDAC inhibits the expression of the BCR.

### Macrophages accumulated in the bone marrow of the *KPC* model show a *V(D)J* recombination pattern

**Fig. 6:**
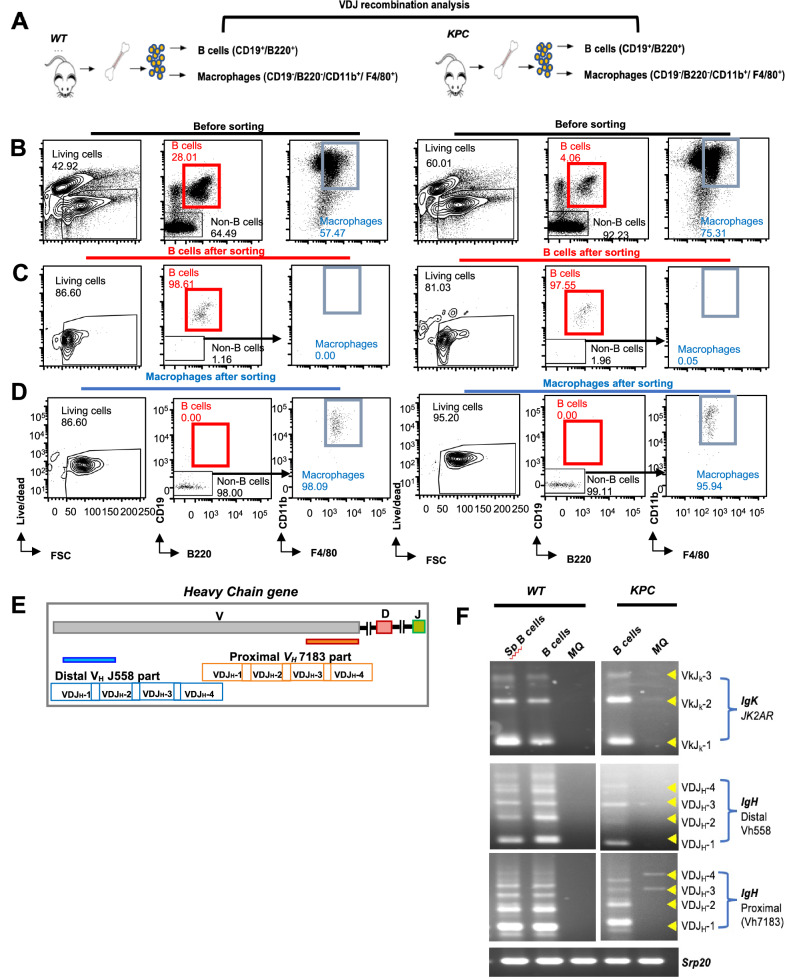
Macrophages in PDAC-bearing mice rearrange BCR genes. **A** Schematic illustration of the experimental design. B cells (CD19^+^B220^+^), and macrophages (CD19^-^B220^-^F4/80^+^CD11b^+^) were FACS sorted from the BM of 8- 12 weeks-old *KPC or wt* mice. Sex-matched mice were used. **B** Gating strategy of FC analysis of B cells (CD19^+^B220^+^) and macrophages (CD19^-^B220^-^F4/80^+^CD11b^+^) from the bone marrow of either *wt* or *KPC* mice before sorting. **C** Dot plots show the purity of the sorted B cells (CD19^+^B220^+^). **D** Dot plots illustrate the purity of the sorted macrophages (CD19^-^B220^-^F4/80^+^CD11b^+^). Representative data of 3 independent experiments per phenotype are shown. Numbers in FC plots indicate the percentages of the cells in the respective gates. **E** Schematic illustration shows the *V(D)J* recombination of proximal *V*_*H*_ *7183* or distal *V*_*H*_ *J558* segment to *DJ*_*H*_*1, DJ*_*H*_*2, DJ*_*H*_*3, or DJ*_*H*_*4* segments of the *IgH* gene locus. **F** Semi-quantitative PCR of *Igk* gene locus rearrangements (*JK2AR* family), *IgH* rearrangements of the distal *VD*_*H*_ gene segments to *J*_*H*_*1, J*_*H*_*2, J*_*H*_*3, and J*_*H*_*4* gene segments (*V*_*H*_*558* family), rearrangements of the proximal *VD*_*H*_ gene segments to *J*_*H*_*1, J*_*H*_*2, J*_*H*_*3, and J*_*H*_*4* gene segments (*V*_*H*_*7183* family). The gDNA input was normalized to *Srp20* PCR products. GDNA isolated from splenic B cells was utilized as a +ve control for *V(D)J* recombination assay.

Next, we conducted additional flow cytometry analysis (Fig. 6A) to identify the non-B cell population (CD19^-^/B220^-^) that comprised 92% and 64% of the bone marrow cells in *KPC* and *wt* mice, respectively. Interestingly, we found that macrophages (CD19^-^B220^-^CD11b^+^F4/80^+^) account for 75% and 57% of the non-B cell population in the *KPC* and *wt* mice, respectively (Fig. 6B). We then questioned whether the macrophages in the *KPC* mice are of B cell origin. To address that, we FACS sorted both B cells (with purities >98% and > 97% in *wt* and *KPC* mice, respectively) and macrophages (with purities >98% and > 95% in *wt* and *KPC* mice, respectively) from the bone marrow of *KPC* and *wt* mice for *V(D)J* recombination analysis (Fig. 6C, D). We examined the recombination pattern of the *Igκ* locus, as well as both the proximal and distal *IgH* segments (Fig. 6E). Intriguingly, only macrophages isolated from the *KPC* mice displayed recombination bands for the proximal *IgH* gene segment (Fig. 6F). This is consistent with the sensitivity of distal *V*_*H*_ to *DJ*_*H*_ recombination to Pax5 expression levels, as it induces large-scale contraction of the distal *V*_*H*_*-DJ*_*H*_ rearrangements of the *IgH* locus [47]. These findings further support our previous observations that macrophages in the context of PDAC may derive from a B cell lineage.

### Detection of highly proliferative Ex-B cell population with an early B cell signature in PDAC

**Fig. 7:**
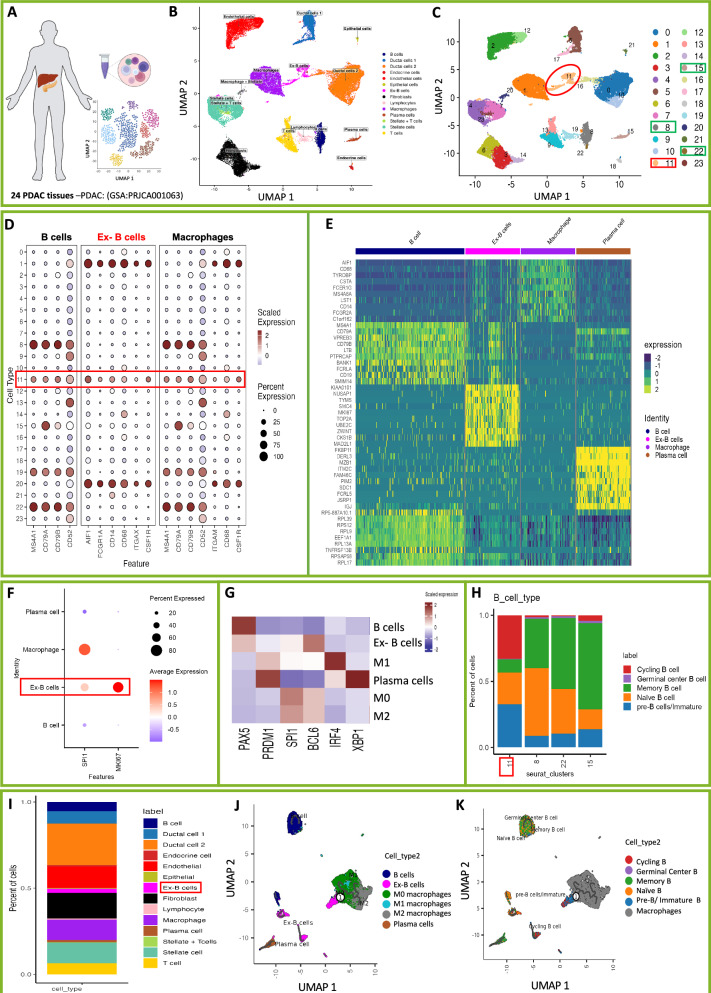
A tumor-infiltrating B cell cluster expresses macrophage markers in human PDAC. **A** Overview of the dataset: single-cell RNA sequencing (scRNA-seq) analysis of 24 PDAC tissues (GSA: PRJCA001063). **B** UMAP visualization of cellular compartments in PDAC tissue. Cells are color-coded based on their assigned cell types, with the corresponding annotations and colours displayed in the legend on the right. **C** UMAP showing detailed 23 distinct cellular clusters identified in PDAC tissues, with clusters 8, 11, 15, and 22 representing B cells. **D** The expression of marker genes for B cells, macrophages, and Ex- B cells clarified the cell type of the clusters. **E** Heatmap showing the expression levels of the top 10 markers in B cell, Ex-B cell, macrophage, and plasma cell clusters. **F** Differential expression analysis of *SPI1 and MKI67* across B cell, Ex-B cell, macrophage, and plasma cell clusters in PDAC tissue. **G** Heatmap of relative expression of the transcription factors *PAX5, PRDM1, SPI1, BCL6, IRF4*, and *XBP1*. Rows represent the cell populations (B cells, Ex-B cells, M1 macrophages, Plasma cells, M0 macrophages, and M2 macrophages). Color intensity reflects scaled gene expression levels, with red indicating higher expression and blue indicating lower expression. **H** Bar plot showing the proportions of different cell types in PDAC. The y axis represents the ratio of pre-B/ immature, naïve, memory, germinal center, and cycling B cells, while the x axis represents the four B cell clusters identified in 24 PDAC tissues. The result identified Ex-B cells (cluster 11) as pre-B/ immature B cells with cycling features. **I** Proportion analysis of different cell clusters identified in PDAC. UMAP visualization of pseudotime and trajectory analysis computed by monocle3. Cells are coloured by cluster (B cell, Ex- B cell, M0 macrophages, M1 macrophages, M2 macrophages, or plasma cells) (**J**), or by B cell developmental subtype (Pre-B/ immature, naïve, memory, germinal centre, and cycling B cells) (**K**).

We analyzed publicly available single-cell RNA sequencing (scRNA-seq) data from 24 PDAC tissues (GSA: PRJCA001063) [52] (Fig. 7A, B). Sub-clustering of the B cell compartment revealed four transcriptionally distinct B cell clusters infiltrating PDAC tissues (Fig. 7C). Interestingly, our analysis identified one B cell subset co-expressing canonical B cell markers (*MS4A1*, *CD79A*, *CD79B*, and *CD52*) along with macrophage-associated markers (*AIF1*, FCGR1A, CD14, *CD68*, and *CSF1R*), suggesting an atypical transcriptional profile (Fig. 7D, E). Notably, we found that *SPI1*, a key transcription factor regulating myeloid lineage commitment, is expressed in the Ex-B cell cluster, but is absent from all conventional B cell clusters. The Ex-B cell cluster also exhibited markedly high expression level of *MKI67*, indicating a high proliferation capacity (Fig. 7F).

Given that Pax5 downregulation is a prerequisite for plasma cell differentiation, we tested whether Pax5 inhibition allows the upregulation of genes required for plasma cell differentiation. For that, we analyzed the expression of key transcription factors involved in B cell- to- plasma cell differentiation, including PRDM1 (Blimp1), BCL6, IRF4, and XBP1. Consistent with our in vitro data, Ex-B cells displayed reduced PAX5 expression, along with decreased expression levels of PRDM1, IRF4, and XBP1 (Fig.7G). Interestingly, Ex-B cells exhibit elevated BCL6 expression, which is known to directly repress Blimp1 [53]. Consistent with our previous observations, Ex-B cells express an elevated level of *SPI1*, the main transcription factor responsible for regulating the macrophage lineage commitment, and is known to be repressed by PAX5 [54]. Thus, downregulation of Pax5 likely relieves this repression, facilitating the acquisition of myeloid features. Overall, the transcriptional profile of Ex-B cells overlaps with that of M2 macrophages, in line with their CD206 expression (*c.f* Fig.3). Developmental stage analysis indicated that these cells predominantly resemble early B cells, ranging from pre-B to immature B cells (Fig. 7H). Quantitative analysis indicates that Ex-B cells comprise a substantial proportion of the tumor- infiltrating B cells (Fig. 7I). Moreover, the Ex-B cell cluster exhibits significant upregulation of genes implicated in cell cycle regulation, mitosis, and DNA repair (e.g., *KIAA0101, NUSAP1, TYMS, SMC4, MKI67, TOP2A, UBE2C, ZWINT, CKS1B, and MAD2L1*). This suggests that these cells are expanding to establish a stable cellular pool with an ongoing dynamic to attenuate the genomic instability occurred during lineage re-programming (*cf* Fig. 7E and Supplementary Fig. 7). Importantly, the cell trajectory analysis identified Ex-B cells to be the only B cell cluster that has direct developmental contact to the TAMs (Fig. 7J, K and Supplementary Fig. 8). Together, these findings highlight a novel mechanism employed by PDAC to evade the immune surveillance, not only by regulating the types of immune cells recruited into the TME, but also by influencing their developmental trajectories and lineage identity.

## Discussion

Tumors can avoid immune detection by acquiring features that hinder anti-tumor immunity. These immune evasion mechanisms are initiated and strengthened during tumor development under immune pressure. While some immunogenic subclones can be efficiently eliminated by anti-tumor T cells. Other cells cannot be eliminated and grow into resistant tumors that are clonally selected and show no response to immune checkpoint blockade therapy. Tumor-infiltrating B cells are an important component in the tumor microenvironment (TME) with diverse functions, including antibody production, antigen presentation, and cellular cytotoxicity [55]. Tumor-infiltrating B cells play an inconsistent role in the TME of PDAC [2, 56]. However, the mechanisms by which the tumor influences the functional fate of B cells remain unclear. In this study, we unveiled a novel immune evasion mechanism employed by PDAC to suppress the anti-tumor activity of B lymphocytes. Our results show that PDAC enforces B cells to tolerate immunosuppressive TME by inhibiting their lymphoid identity and activating the transcription of the myeloid-related genes. It induces B cell reprogramming into functional macrophages with immunosuppressive properties. Interestingly, we detected rapid and dynamic alterations in *the* key surface markers (e.g., CD19, B220, CD79a, CD79b, IgM, and Igk) of B cells cultured under conditions mimicking PDAC. Moreover, these cells exhibited a rapid increase in cell size, adherence, and granularity. Such alterations were correlated to a profound disturbance in the expression of the key transcription factor responsible for maintaining the B cell identity, Pax5.

Pax5 belongs to the highly conserved paired-box (Pax) domain family of transcription factors. Its expression is essential for initiating B cell lineage commitment and is continuously required to maintain the lymphoid identity until its physiological downregulation during the terminal differentiation into plasma cells [16, 33]. Interestingly, Pax5 plays a dual role, acting as a transcriptional activator for B lineage-specific genes and as a repressor for B lineage-inappropriate genes. For example, it activates the transcription of *Cd19*, *Cd79a*, and B cell linker protein (*Blnk*), while suppressing genes like *M-Csf1r*, *Notch1*, and *Flt3* [30]. In this regard, genes regulated by Pax5 were shown to be extremely sensitive to the cellular concentrations of Pax5 [57]. Accordingly, disruptions in Pax5 expression by altered gene dosage or mutations have been correlated with impaired B cell differentiation, as *Pax5*^*–/–*^ B cells were unable to differentiate beyond the pro-B cell stage [22]. Additionally, they show less commitment to the lymphoid lineage fate, as stimulating them with appropriate cytokines induces their de-differentiation into functional macrophages, osteoclasts, granulocytes, natural killer cells, or dendritic cells in vitro [23], and they were able to reconstitute T cells in *Rag2*^*–/–*^ mice [15, 23, 24]. Similarly, our results showed that the B cells that experienced PDAC not only upregulated macrophage-specific genes like *Csf1r, Emr1, Itgam*, and *Cd68*, but also were functionally competent macrophages. It is noteworthy to mention that while almost all the previous reports studied B cell commitment instability utilized *Pax5*^*–/–*^ pro-B cells (with non-rearranged immunoglobulin genes) or similar artificial experimental models, here we show that under PDAC conditions, *Pax5*^*wt*^ B cells from different developmental stages lose the expression of *Pax5* and its B-lineage related genes, retain cellular plasticity, and de-differentiate into functional macrophages. This unique behavior of B cells in PDAC challenges the notion that lineage commitment is a unidirectional process, highlighting that, under certain circumstances, B cells can retain a degree of plasticity that allows them to acquire different lineage identities. Moreover, downregulation of Pax5 permits the induction of Blimp1, the main transcription factor driving plasma cell differentiation [58]. In parallel, BCL6 antagonizes Blimp1 to regulate B cell terminal differentiation into plasma cells [59]. In line with this, our analysis shows that although Ex-B cells downregulated Pax5, plasma developmental stage analysis indicated that these cells predominantly resemble early B cells, ranging from pre-B to immature B cells. Quantitative analysis indicates that Ex-B cells comprise a substantial proportion of the tumor- infiltrating B cells. Moreover, the Ex-B cell cluster exhibits significant upregulation of genes implicated in cell cycle regulation and mitosis.

The question of how *Pax5* is downregulated in *wt* B cells can be partially addressed based on the concept that the developmental commitment to a particular lineage is gradually acquired and maintained by a combination of both cell-intrinsic and extrinsic signals [60]. Accordingly, transcriptional alteration alone is insufficient to trigger lineage reprogramming, unless it is complemented with exposure to appropriate cytokines. For instance, we and others have previously shown that B cells, either express *Pax5* below physiological levels [15] or are entirely deficient [23], can keep the lymphoid identity as long as they are cultured under B cell conditions. That was further supported by a report showing that ectopic expression of *C/EBPa* and *C/EBPb*, but only in the presence of M-CSF, enforces differentiated B cells to undergo efficient reprogramming into macrophages [18]. The authors illustrated that C/EBPs synergize with PU.1 and ETS transcription factors to inhibit the expression of *Pax5*, *Cd19*, and other B cell-related genes, while concurrently activating the expression of *Mac-1* and other myeloid-specific markers [18]. Additionally, our in vitro data showed that PDAC cultures produce elevated levels of M-CSF and GM-CSF relative to the controls. Interestingly, Ex-B cells produce elevated levels of GM-CSF in the CM, compared to the CM of co-culture lacking B cells, suggesting a potential positive feedback loop reinforcing the myeloid characteristics of these cells. In agreement, M-CSF was also found to be significantly elevated in the serum of PDAC patients [61] as well as in the invasive PDAC tissue samples when compared with normal pancreatic tissues [62]. The same study showed that CSF1/CSF1R blockade reprograms the immunosuppressive TAMs and sensitizes the tumor to T-cell checkpoint immunotherapy [62]. In line with these findings, our M-CSF blockade experiments indicate that neutralizing the M-CSF/CSF1R axis partially inhibits B cell reprogramming (data not shown). Previous reports showed that tumor-mediated inflammatory signals disrupt normal cytokine networks and affect hematopoiesis in the bone marrow by prioritizing the production of myeloid cells over lymphocytes [63, 64]. Accordingly, B cell development is severely impaired not only because of competition with myeloid progenitors, but also due to tumor-secreted cytokines [60]. These findings are in complete agreement with our results showing that the bone marrow of the *KPC* mice exhibited heavy accumulation of macrophages, while B cell percentage, absolute cell number, and development were repressed. Interestingly, our data showed that the macrophages isolated from the *KPC* mice have a B cell fingerprint.

Functionally, our data show that the Ex-B cells not only exhibit proficient phagocytic activity but also produce soluble proteins known to be secreted by immunosuppressive TAMs, such as GM-CSF, TNF, CCL20, and CXCL1 [42, 65–67]. Our secretome profiling clearly shows that PSCs were the main source of IL-6, which is known to activate the immunosuppressive phenotype of TAMs and induce PDL-1 expression [68]. In agreement, we detected elevated levels of PDL-1 on the Ex-B cells. Furthermore, GM-CSF–polarized macrophages were shown to release large amounts of TNF [69]. Importantly, we detected significantly elevated levels of attractant molecules such as CCL22, IL-10, and CCL2, which activate macrophage infiltration to the tumor site [70]. Notably, these proteins are also known to promote tumor cell proliferation, invasion, angiogenesis, and metastasis, thereby suggesting a pro-tumorigenic and immunosuppressive nature of the Ex-B cells. For example, CCL2 was found to be upregulated in human and mouse PDAC. It contributes actively to the immunosuppressive TME by inhibiting CD8^+^ T cell infiltration, and its inhibition enhances the sensitivity of PDAC to the immune checkpoint blockade [71]. Furthermore, CCL2 directly inhibits B cell maturation by suppressing the expression and the signaling machinery of the BCR and its co-receptor CD19. Interestingly, both BCR and CD19 expression were restored in *Ccl2*^–/–^ B cells upon antigenic stimulation [72]. This is consistent with our findings showing rapid downregulation of BCR and CD19 on the Ex-B cells. Thus, the overproduction of CCL2 can be interpreted based on its original function in inducing the differentiation of immunosuppressive macrophages, while inhibiting the function and maturation of B cells. Whether blocking CCL2-mediated signaling can restore BCR expression and rescue B cell identity in PDAC needs to be investigated. Finally, it has been previously reported that B cell depletion or deficiency resulted in a reduction in tumor burden of PDAC mouse models, which has been attributed to the elimination of the tumor-promoting functions of B cells. However, based on our findings, we offer an additional explanation that B cell depletion might eliminate an alternative source of the TAMs, which are mostly associated with unfavorable outcomes. Importantly, our bioinformatic analysis revealed that the Ex-B cells detected at the PDAC site exhibited an early B cell transcriptional signature, characterized by the expression of key pre-B cell receptor (pre-BCR) components, including *IGHM, IGLL1*, and *VPREB*. Notably, these cells also displayed a highly proliferative phenotype, with elevated expression of genes associated with DNA repair and mitotic activity, indicating active cell cycling and expansion. Our analysis also revealed that Ex-B cells were the sole B cell cluster that had direct developmental contact with the TAMs. Altogether, our findings combined with earlier studies, lead us to believe that PDAC, among other malignancies, not only affects the tumor-infiltrating B cells but also remotely influences the development, migration, and retention of B cell precursors to the bone marrow [12, 60, 73, 74]. Consequently, dysfunctional B lymphopoiesis is thought to be more than a side-effect of cancer pathology, but rather an important systemic feature of tumor development.

## Materials and methods

### Cell culture

Mouse-derived PDAC organoids and PSCs were cultured as previously described in [29]. PDAC cells were cultured in a 50 µl Matrigel dome in a 24 well plate, at a density of 30 × 10^3^ cells per well, while PSCs were cultivated at a density of 60 × 10^3^ cells per well (ratio 1:2). CM from PDAC organoids, PSCs, or organoids with PSCs were collected, centrifuged at 1800 rpm to remove cells and debris, sterile filtered, and stored at −80 °C for further use. B cells were purified from the bone marrow of *wt* mice using an immunomagnetic negative selection kit (mouse Pan B Cell Isolation Kit II, Miltenyi Biotec, Cat. No. 130-095-813) according to the manufacturer’s protocol. They were then co-cultured with PDAC organoids with or without PSCs for 16 h, or for 5 days in the CM of the respective cells. Macrophage culture was established by growing murine BM-derived *wt* cells in a medium supplemented with 20 ng/ml M-CSF.

### Mouse samples

Samples from the PDAC mouse models: *KPC* [50] and *KC* [51] used for IF staining, flow cytometry analysis, or *V(D)J* recombination analysis were kindly provided by Prof. Thomas Wirth. Littermates carrying the same genotype but not expressing Cre recombinase were used as controls and were designated as WT mice. The *KC* mice had been treated with cerulein, as described in [51], to induce acute pancreatitis and later on pancreatic cancer. Pan-RFP mice carrying constitutively active *ROSA26-tdRFP* alleles (referred to in the manuscript as *Ac-RFP* mice) were obtained from Professor Hartmut Geiger (Institute of Molecular Medicine, Ulm University) and were previously generated by breeding *C57BL/6-Gt(ROSA)26Sortm1Hjf/Ieg* mice [75] with animals from a germline Cre-deleter strain [76]. Offspring with irreversibly activated ROSA26-driven fluorescent tdRFP reporter as a result of loxP/Cre-mediated recombination in the germline were backcrossed for >10 generations onto C57BL/6, therefore removing the Cre recombinase transgene. Only homozygote *Ac-RFP* mice were used [48].

### Human tissue samples

Human PDAC patient tissue samples were identified and obtained from the Clinic of General and Visceral Surgery, University Hospital of Ulm.

### Flow cytometry analysis

Single-cell suspensions were incubated for 20 min on ice with CD16/CD32 antibodies (BD Bioscience- 553141) to block Fc receptors on the cell surface. Dead cells were excluded by Fixable Viability Dye staining. Antibody dilutions were determined for surface or intracellular staining according to the manufacturer’s instructions. For extracellular staining, cells were resuspended in the pre-diluted antibodies and kept on ice for 20 min. The unbound antibodies were then washed out by PBS + 5% FBS. Antibodies used for extra- or intracellular staining are listed in (Table S1). Intracellular stainings were performed using Fix and Perm cell permeabilization Kit (ThermoFischer Scientific, Cat. No. GAS004) (Table S2) according to the manufacturer’s instructions. Stained cells were acquired at a MACSQuant 10 Analyzer flow cytometer (Miltenyi Biontech). In general, numbers in the dot plots refer to the percentages of cells in the respective gates, and numbers in the histograms show the mean fluorescence intensity.

### Enzyme-linked immunospot assay (ELISpot)

ELISpot assays were performed according to the manufacturer’s instructions to quantify the number of IgG- or IgM-secreting B cells. ELISpot plates containing Immobilon-P membranes (Mabtech, Cat. No. 3654-WP-10) were activated with 70% ethanol and coated with anti-IgG (Mabtech, Cat. No. 3825-2 A) or anti-IgM (Mabtech: 3885-2 A). Seeded cells were incubated for 24 h at 37 °C. Biotinylated anti-IgM and Streptavidin-ALP or anti-IgG Streptavidin-ALP were added to the wells to detect antigen-specific IgM or IgG. The plates were incubated, washed, and finally developed with filtered BCIP/NBT-Plus until distinct spots appeared. ELISpot data were normalized to the number of cells seeded per well. Spot counts were converted to spots per 1 × 10^5^ cells. For that, we used the formula: (spots counted ÷ cells plated) × 10⁵. Normalized values were used for statistical analysis.

### Retroviral transfection of Phoenix cells

Phoenix cells were used as a retrovirus producer cell line. A day before transfection, 2.5–3 × 10^5^ cells were seeded in RPMI medium. The cells were incubated till they reached 60–70% confluency. Directly before the transfection, the medium was replaced with 1 mL fresh RPMI medium. For cell transfection, GeneJuice (Millipore) was mixed with RPMI medium and incubated for 5 min at RT. Afterward, 1 μg of the pMIG-*Pax5* plasmid was added, mixed by vortexing and incubated for 15 min at RT. Finally, the mixture was added to the phoenix cells. Transfected cells were incubated at 37 °C. The supernatant was harvested after 48 h, then filtered through a 0.45 μm filter. The retroviral supernatant was either freshly used for transduction or stored at -80 °C until further use.

### Retroviral transduction of B cells

For transduction, 10 μg/mL polybrene was added to the filtered retroviral supernatant, mixed by vortexing and incubated for 5 min at RT. Thereafter, the cell pellet of 2.5–3 × 10^5^ B cells was resuspended in 0.5–1 mL of retroviral supernatant supplemented with polybrene. The cells were then centrifuged at 1800 rpm, 3 h at 37 °C. Finally, the supernatant was carefully aspirated, and the cell pellet was resuspended in fresh RPMI medium. Transduction efficiency was determined by flow cytometry to monitor *Pax5*-GFP.

### T cell- Ex-B cell co-culture

B cells were isolated and cultured under PDAC conditions, allowing the trans-differentiation. On day five, CD3^+^CD8a^+^ T cells were isolated from the spleens of *wt* C57BL/6 J mice using a CD8a^+^ T cell isolation kit (Miltenyi Biotec, Cat. No.130-104-075) according to the manufacturer’s instructions. Depending on the experimental condition, the T cells were stimulated using mouse T-activator CD3/CD28 Dynabeads (Thermo Fisher Scientific, Cat. No.11456D) according to the manufacturer’s instructions. T cells were resuspended in the supernatant of the corresponding condition and added back into the B cell culture in ratios of 1:1, 2:1, 4:1 (B: T cell).

### Phagocytosis assay

0.5 × 10^6^ B cells were cultured in the CM of PDAC or PDAC & PSCs for 5 days to allow their re-programming into macrophages. The phagocytic capacity was then tested by adding Zymosan labeled for fluorescence at Ex/Em 540/570 nm (Abcam, Cat. No. ab234054, red Zymosan) (Table S2). The cells were incubated at 37 °C with Zymosan for 2–3 h. The cells were then washed three times with pre-warmed assay buffer to remove any non-engulfed Zymosan. The phagocytic capacity of the cells has been analyzed by fluorescent microscopy.

### Cytokine/chemokine array analysis

Supernatants from different co-cultures were harvested, centrifuged at 1800 rpm to remove cells and debris, and sterile filtered through 0.2 µm strainers. Aliquots were stored at -80°C until further analysis. The Olink target 96 platform was used to detect soluble proteins produced in the CM of different co-culture setups. Cell-free media were used to subtract the background.

### Multiplex immunofluorescence staining (MIF)

Multiplex IHC/IF (MIF) staining with IF detection is performed using the Opal 7-Color Manual IHC kit (Akoya Biosciences, Cat. No. NEL811001KT) according to the manufacturer’s instructions. FFPE sections (Formalin-Fixed Paraffin-Embedded) were deparaffinized, and antigens were separated by heat-induced epitope retrieval with citrate buffer (pH 6) or Tris/EDTA (pH 9). Each section was subjected to several successive rounds of staining; each round included blocking of endogenous peroxidase and blocking of non-specific proteins, followed by primary antibody and corresponding secondary Horseradish peroxidase-conjugated polymer. Each Horseradish peroxidase-conjugated polymer mediates the covalent binding of different fluorophores using tyramide signal amplification. This covalent reaction is followed by additional antigen retrieval in heated citric acid buffer (pH 6) or Tris/EDTA (pH 9) for 10 min to remove the antibodies before the next round of staining. After all successive staining reactions, the sections are counterstained with DAPI.

### *V(D)J* recombination analysis

Purified CD19^+^/B220^+^ bone marrow-derived B cells cultured under different conditions were collected and pelleted by centrifugation. Genomic DNA was extracted from the cell pellet using the Puregene Kit (Qiagen, Cat. No. 158023) following the manufacturer’s instructions. Semi-quantitative real-time PCR was performed to analyze immunoglobulin *heavy* and *light chain* genes, using the primer sequences listed in (Table S3), as described previously in [47]. Gradient PCR was employed to determine optimal annealing temperatures. Amplified PCR products were visualized by agarose gel electrophoresis.

### Quantitative Real-time PCR (qRT-PCR)

Purified B cells were cultured under different conditions for 16 h or 5 days. RNA was extracted by using the ReliaPrep RNA Cell Miniprep System (Promega, Cat. No. Z6012) according to instructions provided by the manufacturer. Residual genomic DNA was digested using DNase I (ThermoFisher Scientific, Cat. No. EN0521). cDNA synthesis was performed with the RevertAid First Strand cDNA Synthesis Kit (ThermoFisher Scientific Cat. No. K1662) as recommended by the manufacturer. Sequences of oligonucleotides used for the qRT-PCR are listed in (Table S4). QRT-PCR was conducted using SYBR Green PCR Master Mix (ThermoFischer Scientific, Cat. No. 4309155) to quantify gene expression levels.

### ScRNA-seq data analysis

For the single-cell analysis, data from 24 PDAC tissues were used from GSA: PRJCA001063. Seurat v.5.0.1 was used for all analyses. Briefly, we input the RNA counts from the filtered_feature_bc_matrix.h5 output from Cell Ranger from 10x Genomics and added the RNA and ATAC Assay into a Seurat object using the “CreateSeuratObject” functions. A series of quality filters was applied to only include the barcodes that fell into the categories recommended by Seurat: nCount_RNA < 5000 & nCount_RNA > 1200, and to avoid assaying possible dead cell or a sign of cellular stress and apoptosis with too high a proportion of mitochondrial gene expression over the total transcript counts, we limited the mitochondrial content percentage (percent.mt) < 10. The RNA data were then scaled and normalized using Seurat’s “NormalizeData” with normalization. method = “LogNormalize” and scale.factor = 10000, FindVariableFeatures with selection.method = “vst”, and nfeatures = 3000. Data was then scaled using “ScaleData” and the top 50 PCA dimensions were assayed via “FindNeighbors” and “FindClusters” (with parameters: resolution = 0.6 and dims=1:30) functions. The “RunUMAP” function (with default parameters) and the first 50 PCs were used to perform the Uniform Manifold Approximation and Projection (UMAP), a standard dimensional reduction step, to visualize the snRNA data. The data was then batch corrected using “RunHarmony”, with max.iter.harmony = 20, and using the same dims and resolution for “FindNeighbors” and “FindClusters” and “RunUMAP” on the merged harmony corrected object. For cell type annotation, the Seurat’s “AddModuleScore” function was used by scoring the gene sets from Peng et al (ref). These gene sets were: Ductal cell 1 markers include AMBP, CFTR, and MMP7. Ductal cell 2 is characterized by KRT19, KRT7, TSPAN8, and SLPI. Acinar cells express PRSS1, CTRB1, CTRB2, and REG1B. Endocrine cells include CHGB, CHGA, INS, and IAPP. Stellate cell markers are RGS5, ACTA2, PDGFRB, and ADIRF. Fibroblasts are marked by LUM, DCN, and COL1A1. Endothelial cells express CDH5, PLVAP, VWF, and CLDN5. Macrophages are identified by AIF1, FCGR1A, CD14, CD68, ITGAX, and CSF1R. T cells express CD3D, CD3E, CD4, CD8A, and CD8B. B cells are marked by MS4A1, CD79A, CD79B, and CD52. Epithelial markers include EPCAM, CDH1, CLDN3, and CLDN4. A B cell population with macrophage markers (Ex-B cells) includes MS4A1, CD79A, CD79B, CD52, ITGAM, CD68, and CSF1R. Monocytes express ITGAM, CD14, and HLA-DRA, are negative for MRC1 and CD86, and positive for the rest. Plasma cells express TNFRSF17 and SDC1. Dendritic cells include ITGAX, HLA-DRA, and ITGAM. NK T cells are characterized by NCAM1, CD3D, CD3G, and CD3E. Neutrophils express FCGR3A, CEACAM8, and FUT4.

### Cytolution analysis

The AI-based Cytolution tool was used to re-analyze the FC data to generate the UMAPs in Fig. 4. FCS files were split into two groups: *KPC* and *WT*. Batch effect correction was performed using the CytoCorrectV1 algorithm. Compensation was adjusted manually. The data quality was checked, and data cleaning was performed using the CytoV2 and SingletV2 algorithms. Further post-step sampling was disabled. Outliers were detected by the CytoTime algorithm (number of iterations = 2 for spillover calculations) and excluded. Duplets and dead cells were excluded via FSC and SSC, and the gating for Zomby Aqua negative events. For data transformation, the AsinH transformation was used with Cyto v4, calculating optimal transformation parameters for each channel (parameter version 1.1.0). A cutoff definition (based on settings by Cutoff Estimator Version 2.0) was performed to define positive and negative as well as low and high events for each channel. The population definition was performed by creating a population manually using the assisted tree builder. In this step, clustering was performed using the Parc algorithm version 2.2 and using Parc version 2.0 with high resolution for fine clustering. For results visualization and dimensionality reduction, UMAPs were created.

### Statistical analysis

The results are presented as the mean with each individual data point or in a bar graph ± SEM. GraphPad Prism (version 6) software was used for statistical analysis. Data distribution was examined using the D’Agostino & Pearson omnibus test. If the normality test could not be applied, due to a small sample size, the data were treated as “not normally distributed”. *P* values were calculated using the statistical tests mentioned in the figure legends. A *P*-value less than 0.05 was considered significant. (****P < 0.0001, ***P < 0.001, **P < 0.01, *P < 0.05, and n.s. = not significant).

### Animal approval

All animal procedures were conducted at Ulm University under approved institutional guidelines and in accordance with the German Animal Welfare. The mice were kept at the animal facility of the University of Ulm. Collection of wildtype cells and tissues for this study was approved under the internal registration number o.238-2.

## Supplementary information

Supplementary Figures

Supplementary Tables

Uncropped Figures

## Data Availability

We re-analyzed an existing, publicly available scRNA sequencing dataset of human PDAC, sourced from GSA: PRJCA001063 in the Genome Sequence Archive. All R-scripts and code used for data processing and analysis are available at: https://github.com/Hend2468/PDAC-induces-B-cell-reprogramming.
