# Supplementary material for: Pancreatic cancer induces B cell lineage plasticity via Pax5 inhibition to sustain immunosuppression
Source: Cell Death Discov. 2026 Jun 2;12:265. doi: 10.1038/s41420-026-03174-z (PMC13230850; doi:10.1038/s41420-026-03174-z)
Supplement: Supplementary file 1 — Supplementary Figures [file 41420_2026_3174_MOESM1_ESM.pdf]

Supplementary Figure 1: Cellular components used for the 3D co-culture systems

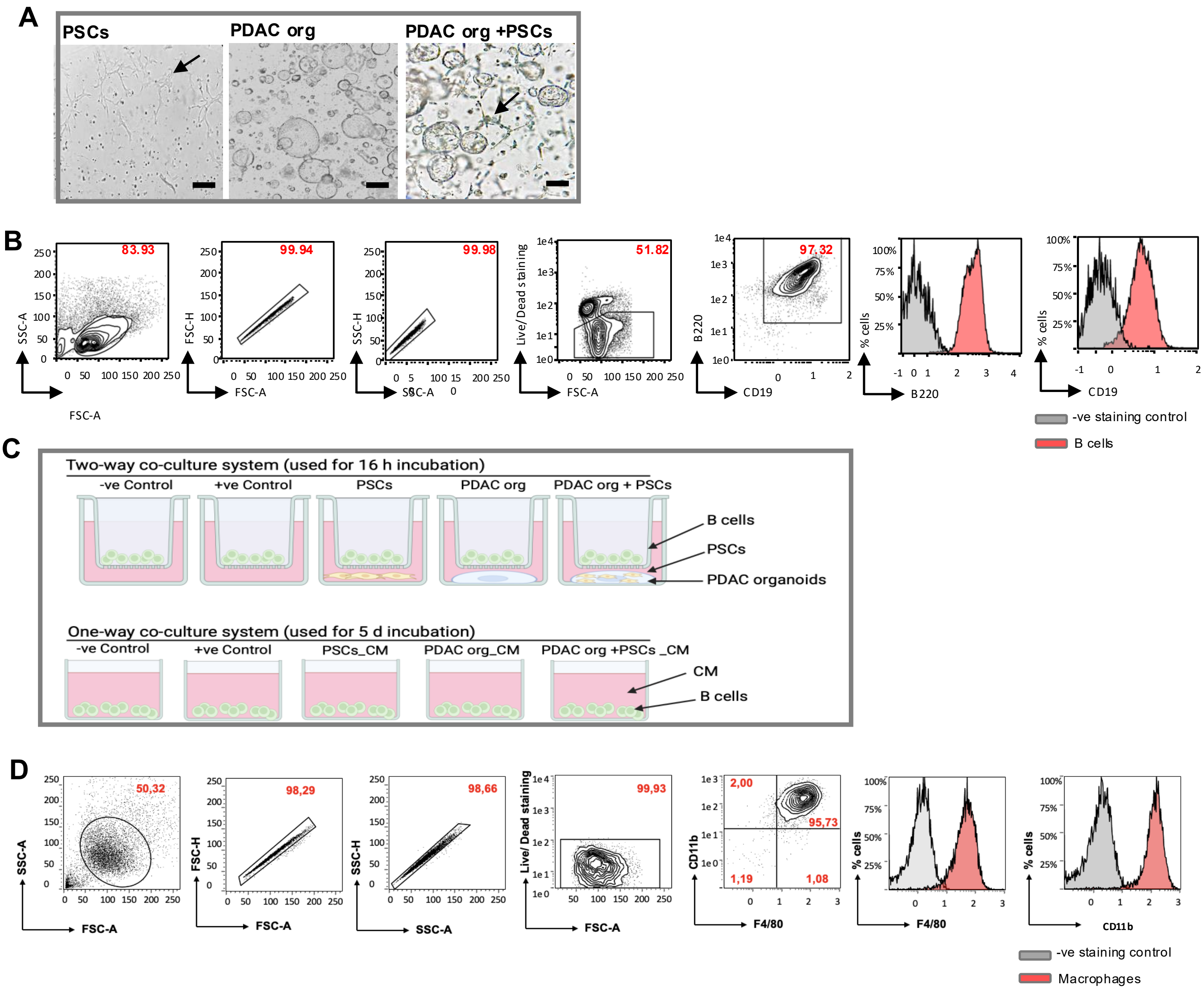

Supplementary Figure 1 | 3D co-culture of PDAC components and pure B cell and macrophage culture.
A) White field microscopy pictures of successful 3D culture of PSCs (left), PDAC mouse organoid (middle), and PDAC mouse organoid & PSCs (right). **B)** FC analysis of the $CD19^{+}B220^{+}$ purified bone marrow- derived B cells used for the co-culture experiment. The FC plots shows the gating strategy to identify the $CD19^{+}B220^{+}$ B cells. FC histograms of B220 (left), and CD19 (right) expression on purified B cells, in comparison to -ve staining controls. **C)** Graphical illustration of the two different co-culture systems: 1- Two-way co-culture system (short-term culture) was used to culture B cells in 1µm trans-well membranes with PSCs, PDAC organoid or PDAC organoid with PSCs for 16 h (upper panel). 2- One-way co-culture system (long-term culture) was used to culture B cells in sterile filtered conditioned media (CM) of PSCs, PDAC organoid with or without PSCs for 5 days (lower panel panel). **D)** Representative FC plots of bone marrow- derived cells cultured in cell culture medium supplemented with M-CSF for 5 d to generate pure macrophage culture. The FC plots shows the gating strategy to identify the CD11b+/F480+ macrophages. FC histograms of F4/80 (left), and CD11b (right) expression on macrophages, in comparison to -ve staining controls.

Supplementary Figure 2: PDAC- experienced B cells reprogrammed into Macrophages

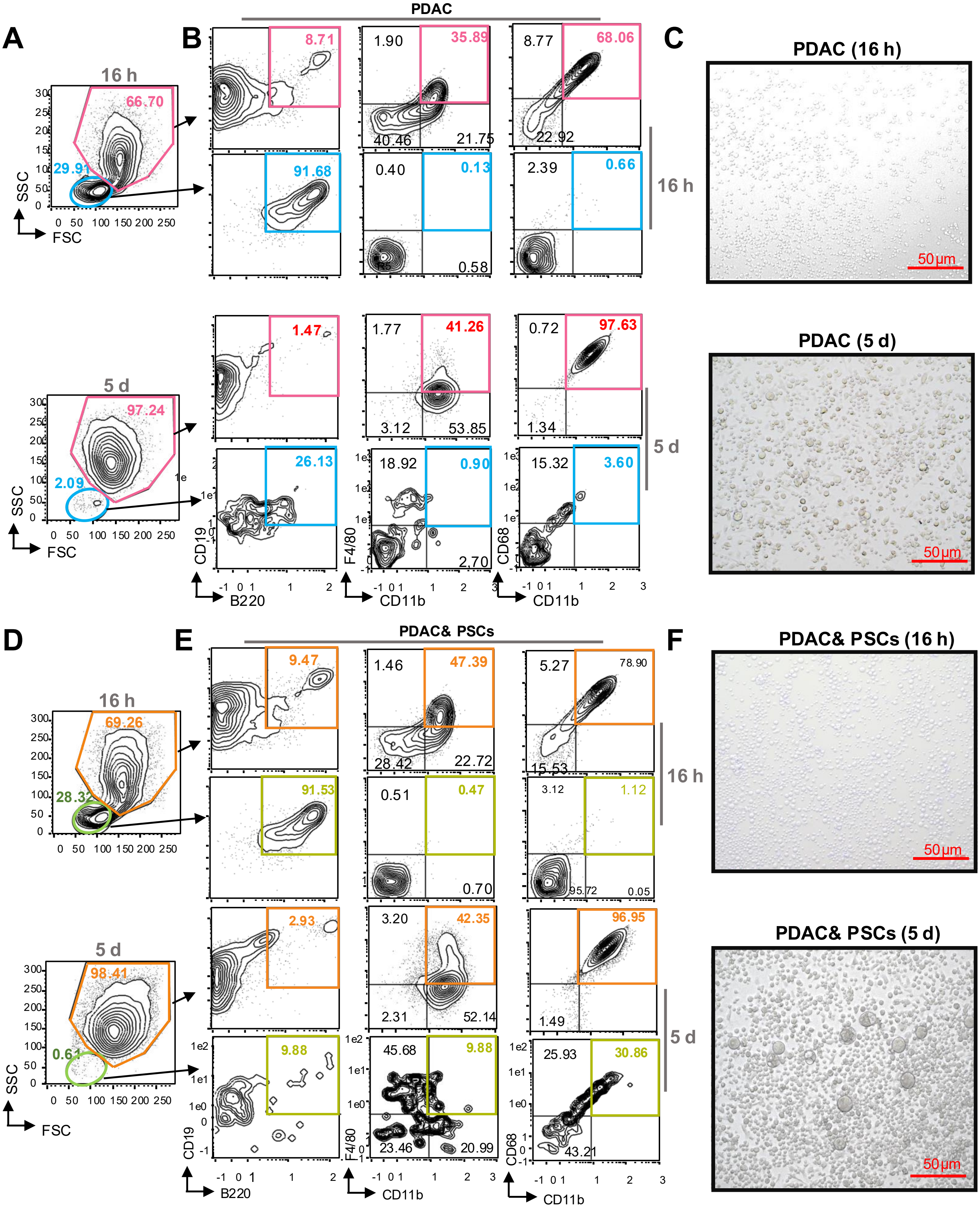

Supplementary Figure 2 | PDAC- experienced B cells reprogrammed into Macrophages

A) FC analysis of FSC vs. SSC plots of B cells co-cultured for 16 h (upper panel) or 5 d (lower panel) with PDAC mouse organoid. **B)** FC plots of B cells co-cultured for 16 h (upper panels) or 5 d (lower panels) co-cultured with PDAC mouse organoids showing the expression of CD19/B220 (left panel), F4/80/CD11b (middle panel), and CD68/CD11b (right panel). **C)** Representative bright field microscopic pictures for 16 h- old two-ways culture (upper) or 5 d- old one-way culture (lower) of bm- derived B cells in the CM of PDAC mouse organoid. 20× magnification, 50 µm size bar. **D)** B cells co-cultured with PDAC& PSCs for 16 h (upper panel) or 5 d (lower panel). **E)** FC analysis showing the expression of CD19/B220 (left panel), F4/80/CD11b (middle panel), and CD11b/CD68 (right panel) of B cells co-cultured for 16 h (upper panels) or 5 d (lower panels) co-cultured with PDAC& PSCs. Numbers in density plots indicate the percentages of cells in the respective gates. **F)** Representative bright field microscopic pictures for 16 h- old two-ways culture (upper) or 5 d- old one-way culture (lower) of bm- derived B cells in the CM of PDAC& PSCs. 20× magnification, 50 µm size bar

Supplementary Figure 3: Immunosuppression in PDAC TME

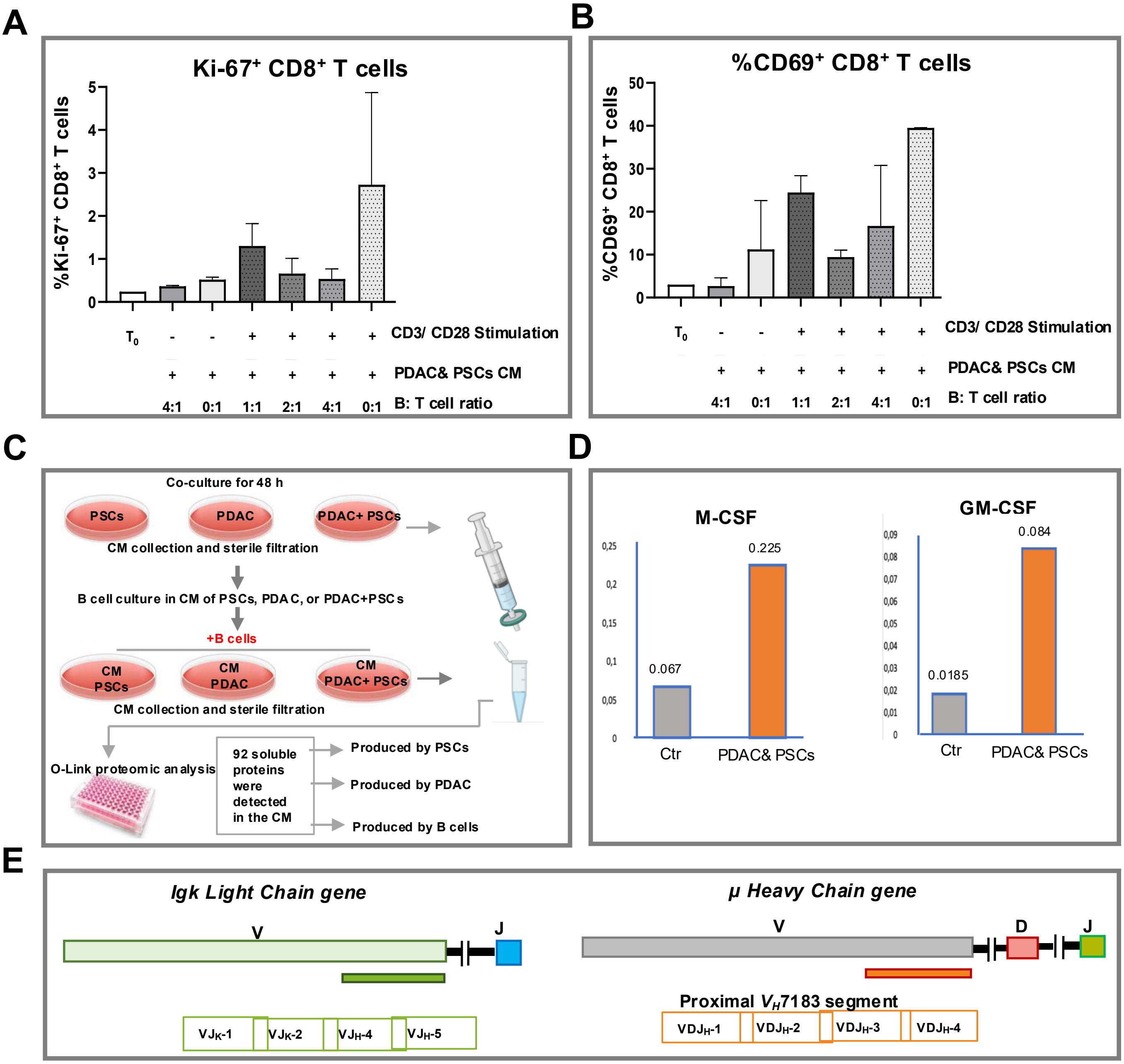

Supplementary Figure 3 | Immunosuppression in PDAC TME

Bar graph of FC data showing the percentage of **A)** The proliferation marker Ki-67⁺ cells (n= 2), or **B)** **T**he early activation marker CD69⁺ cells (n= 2) among CD3⁺CD8⁺ T cells stimulated with CD3/CD28 beads. Freshly isolated CD3⁺CD8⁺ T cells were used as a timepoint zero control (T_0). T cells cultured in PDAC& PSCs CM without Ex-B cells served as an additional control. T cells were stimulated with CD3/CD28 beads or left unstimulated and co-cultured with Ex-B cells in different ratios as indicated in the figure. **C)** Simplified illustration showing the experimental design of secretome analysis. CM of PSCs, PDAC organoids, PDAC& PSCs were collected after 48 h of culture. CM were either sterile filtered before using them for the secretome analysis or for B cell treatment. B cells were treated with the CM for 24 h and the CM were then filtered and used for the analysis. **D)** Production of M-CSF (left) or GM-CSF **(right) in the** control medium or in the CM of PDAC& PSCs culture**. E)** Schematic illustration of the VJ recombination in the *IgK* light chain gene locus (left panel) and V(D)J recombination in the *μ heavy chain* gene locus (right panel).

Supplementary Figure 4: RFP tracing of PDAC-induced B cell reprogramming

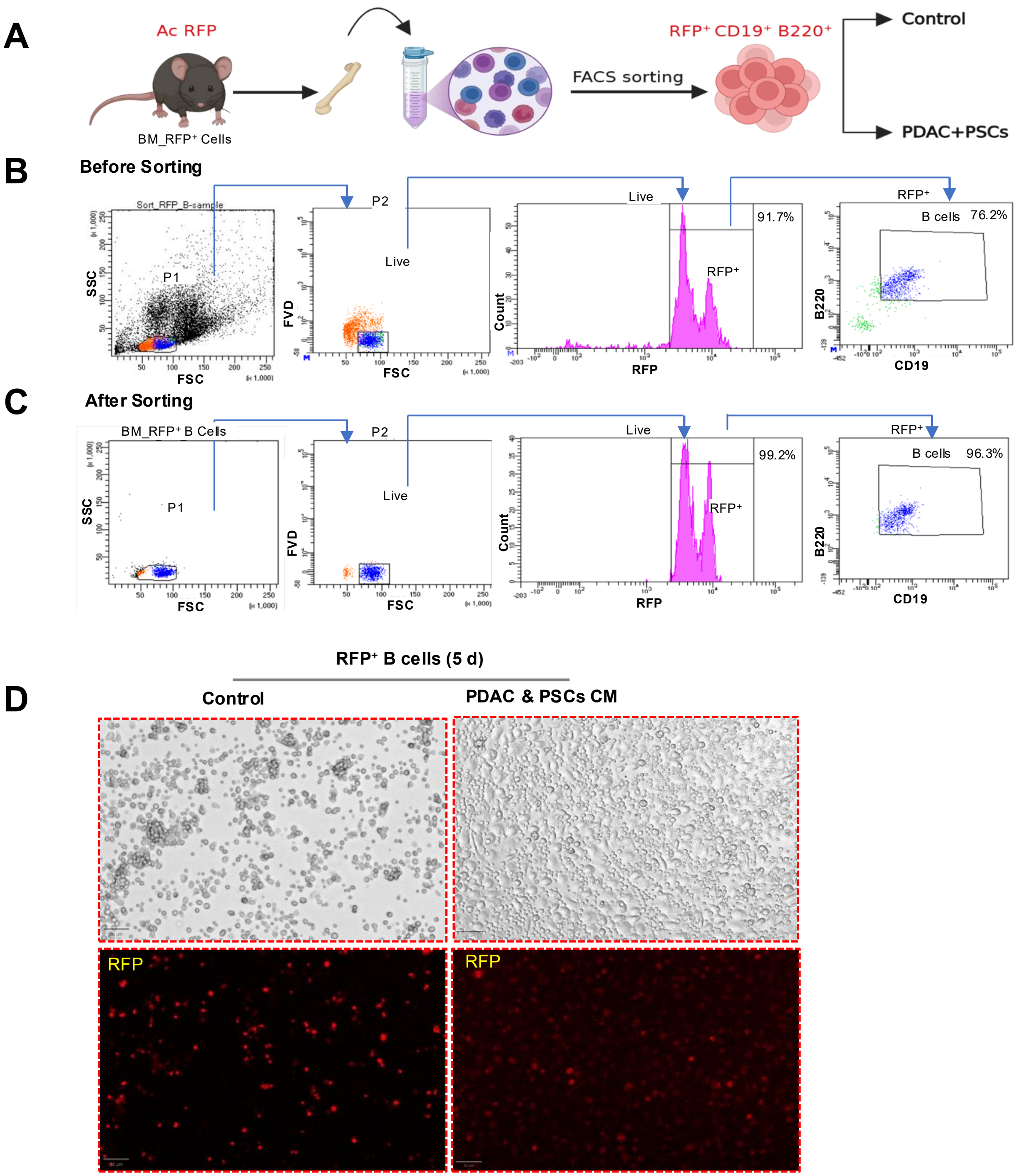

Supplementary Figure 4 | PDAC induces RFP⁺CD19⁺B220⁺ B cells reprogramming
A) Schematic illustration of the experimental setup: BM- derived cells isolated from the Pan *RFP* mouse line (*Ac-RFP*). **B)** Gating strategy used for the sorting of RFP⁺CD19⁺B220⁺ B cells (before sorting). **C)** Gating strategy used to confirm the purity of the RFP⁺CD19⁺B220⁺ B cells (after sorting). **D)** RFP⁺CD19⁺B220⁺ sorted B cells were either cultured under standard B cell conditions or under PDAC conditions (PDAC& PSCs). Bright field (upper panel), or RFP florescent (lower panel) images of RFP⁺CD19⁺B220⁺ B cells cultured for 5 d under the indicated conditions.

Supplementary Figure 5: **Ectopic expression of Pax5 rescues B cell identity but induces cell death under PDAC conditions**

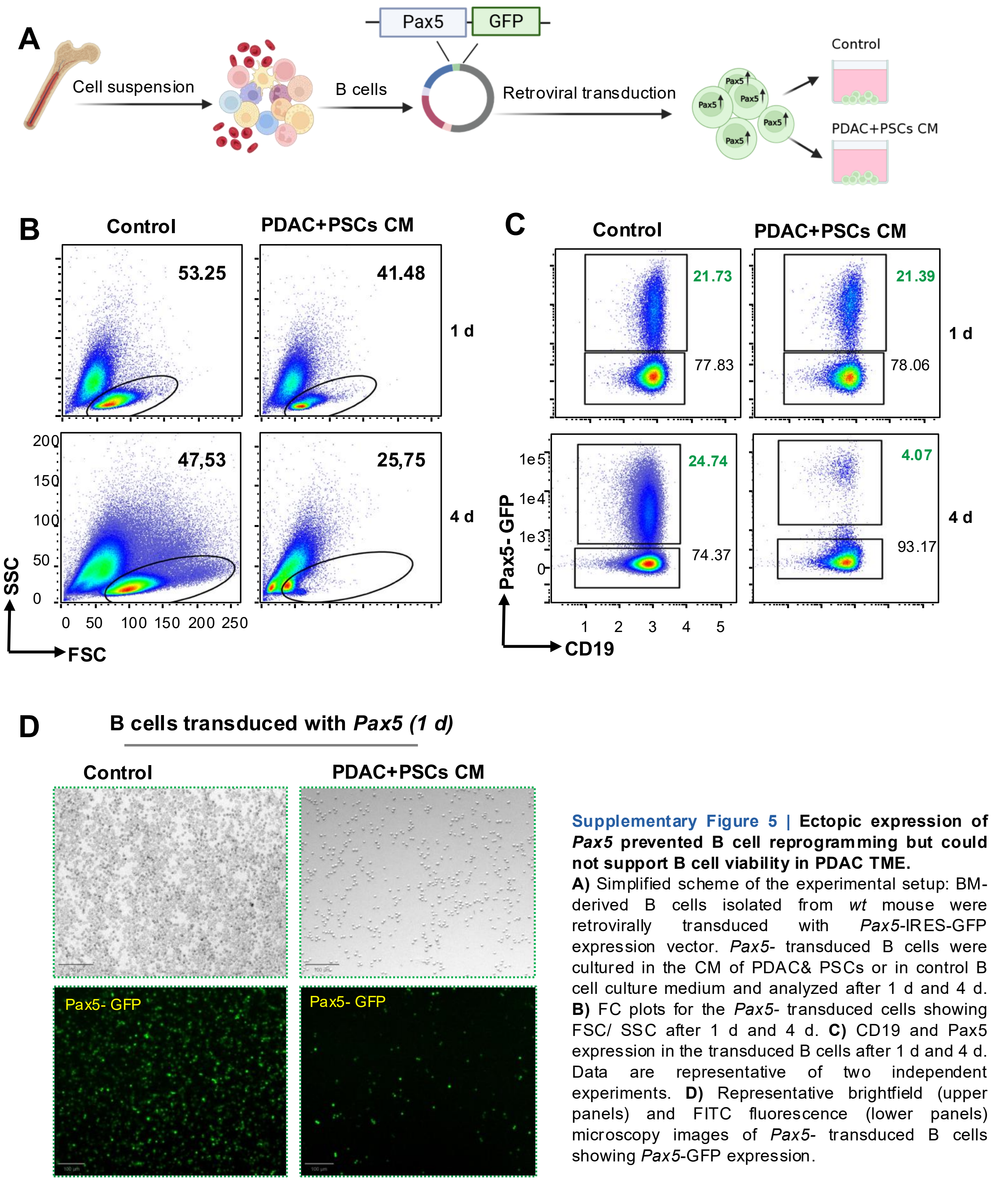

Supplementary Figure 5 | Ectopic expression of *Pax5* prevented B cell reprogramming but could not support B cell viability in PDAC TME.
A) Simplified scheme of the experimental setup: BM-derived B cells isolated from *wt* mouse were retrovirally transduced with *Pax5*-IRES-GFP expression vector. *Pax5*- transduced B cells were cultured in the CM of PDAC& PSCs or in control B cell culture medium and analyzed after 1 d and 4 d. **B)** FC plots for the *Pax5*- transduced cells showing FSC/ SSC after 1 d and 4 d. **C)** CD19 and Pax5 expression in the transduced B cells after 1 d and 4 d. Data are representative of two independent experiments. **D)** Representative brightfield (upper panels) and FITC fluorescence (lower panels) microscopy images of *Pax5*- transduced B cells showing *Pax5*-GFP expression.

Supplementary Figure 6: B cells in dense aggregations are protected from lineage re-programming

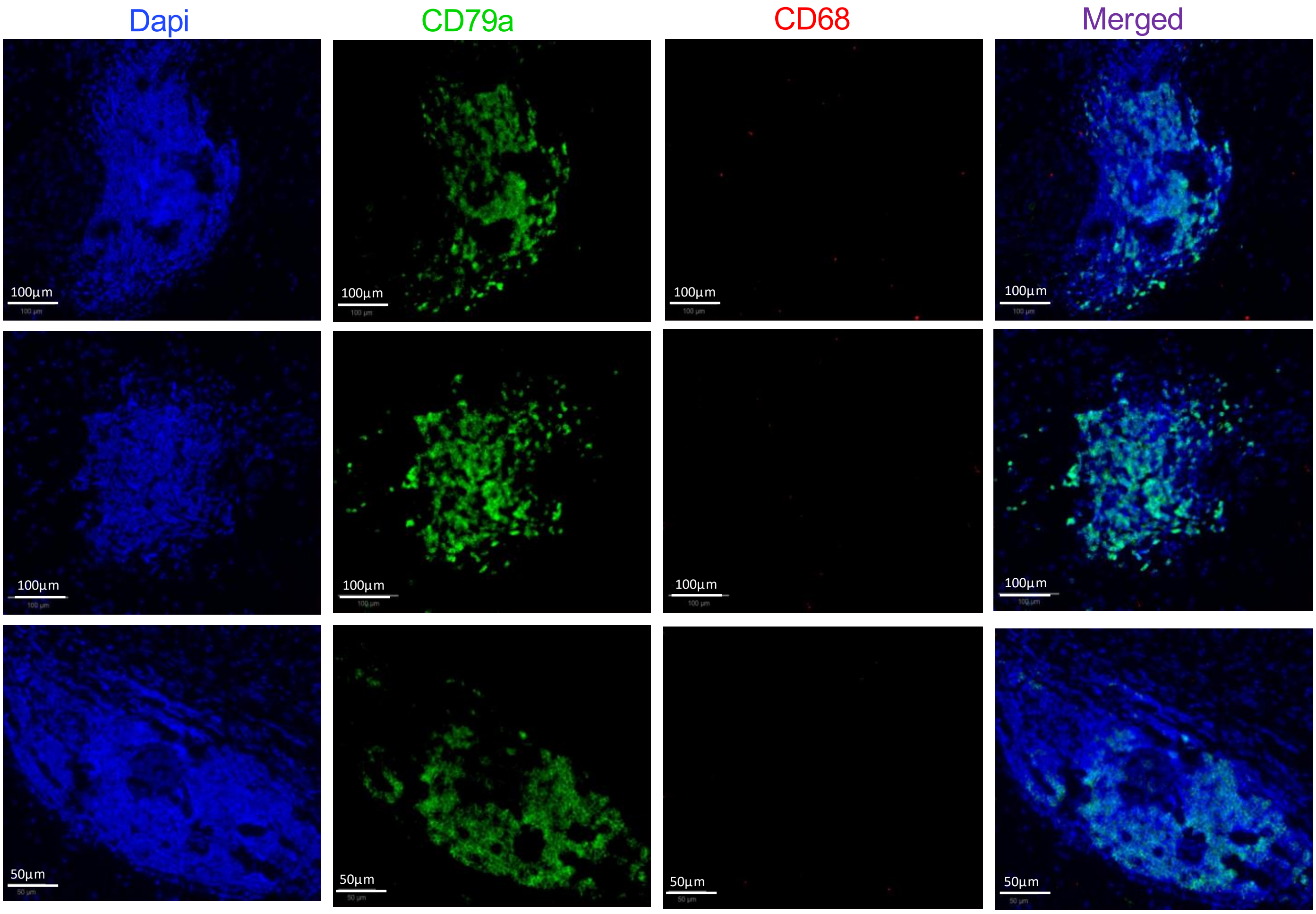

Supplementary Figure 6 | B cells in large aggregations are protected from the transdifferentiation
Representative IF staining of formalin- fixed paraffin- embedded PDAC tissue sections from different patients of CD79a (green) and CD68 (red) in large B cell aggregations. Scale bars in the upper two panel: 100µm, and in the lowest panel: 50µm.

Supplementary Figure 7: Ex-B Cells Exhibit Activation of Cell Cycle and DNA Repair Pathways

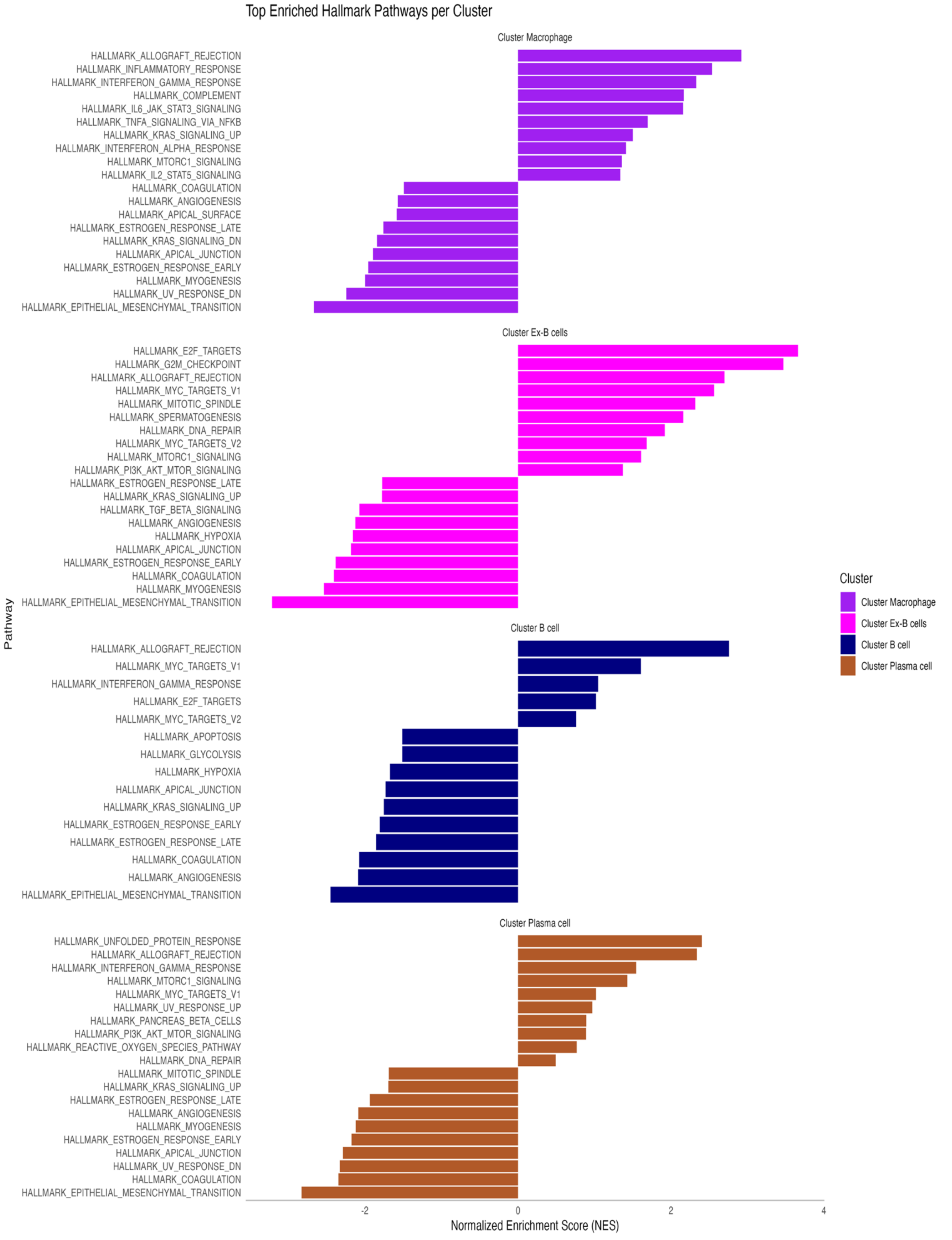

Supplementary Figure 7 | Ex-B cells show activation in pathways involved in cell cycle and DNA repair.
Bar plot illustrating the top enriched hallmark pathways, ranked by gene set variation analysis scores, comparing Ex-B cells, macrophages, B cells, and plasma cells. Up- and down-regulated signalling pathways are shown to right and left side, respectively.

Supplementary Figure 8: Identification of different TAM clusters infiltrate PDAC tissue

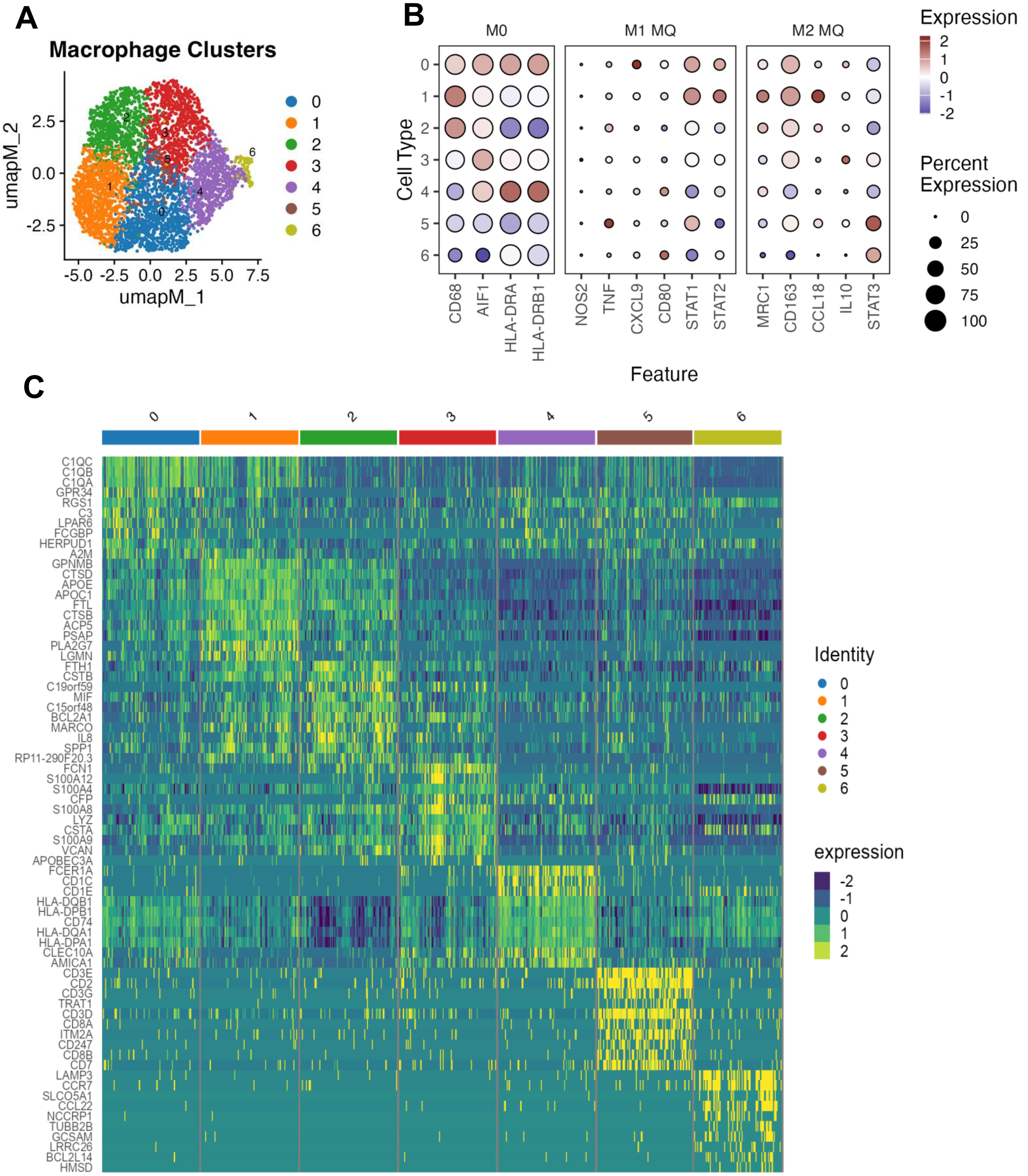

Supplementary Figure 8 | Different TAMs infiltrate PDAC tissue.
A) UMAP visualization of six different TAMs clusters infiltrate PDAC tissue. **B)** The expression of marker genes for six different clusters represent M0, M1, and M2 TAMs. **C)** Heatmap showing the expression levels of top 10 markers in different TAM clusters.
