# Supplementary material for: Pancreatic cancer induces B cell lineage plasticity via Pax5 inhibition to sustain immunosuppression
Source: Cell Death Discov. 2026 Jun 2;12:265. doi: 10.1038/s41420-026-03174-z (PMC13230850; doi:10.1038/s41420-026-03174-z)
Supplement: Supplementary file 2 — Supplementary Tables [file 41420_2026_3174_MOESM2_ESM.docx]

**Supplementary Table 1**

| **List of mouse antibodies** | | | | | | |
| --- | --- | --- | --- | --- | --- | --- |
| **Name** | **Fluorochrome** | **Host** | **Isotype** | **Clone** | **Company** | **Cat. Nr.** |
| CD19 | Percp.Cy5.5 | Rat | IgG2aκ | 1D3 | BioLegend | 561113 |
| CD19 | FITC | Rat | IgG2a | 6D5 | BioLegend | 115506 |
| CD11b | PE | Rat |  | M1/70 | eBioscience | 12-0112-82 |
| CD11b | PE-Cy7 | Rat | IgG2bk | M1/70 | eBioscience | 25-0112-81 |
| CD11b | APC-eFluor780 | Rat | IgG2b | M1/70 | eBioscience | 47-0112-82 |
| IgM | FITC | Goat | IgG2b | Polyclonal | SouthernBiotech | 1021-02 |
| IgM | APC | Rat | IgG2b | 1B4B1 | SouthernBiotech | 1140-11 |
| IgD | APC | Rat | IgG2a | 11-26c | Invitrogen | 17-5993-80 |
| IgD | PE | Rat | IgG2a | 11-26 | SouthernBiotech | 1120-09 |
| CD68 | APC Vio770 | Human cell line | IgG1 | REA835 | Miltenyi biotec | 130-112-859 |
| F4/80 | eF450 | Rat | IgG2aκ | BM8 | eBioscience | 48-4801-82 |
| F4/80 | PE | Human cell line | IgG1 | REA126 | Miltenyi biotec | 130-116-499 |
| F4/80 | AF647 | Rat | IgG2a | BM8 | BioLegend | 123122 |
| IgK | PE | Goat | IgG | Polyclonal | SouthernBiotech | 1050-09 |
| IgK | APC-Vio770 | Human cell line | IgG1 | REA879 | Miltenyi biotec | 130-114-403 |
| PD-L1 | PE | Rat |  | 10F.9G2 | BD Bioscience | 568085 |
| CD45R(B220) | Vioblue | Human cell line | IgG1 | REA755 | Miltenyi biotec | 130-110-851 |
| CD45R(B220) | PE-Cy7 | Rat | IgG2ak | RA3-6B2 | Invitrogen | 25-0452-81 |
| FcBlock(CD16/CD32) | - | Rat |  | 2.4G2 | BD Bioscience | 553141 |

**Supplementary Table 2**

| **List of Kits** | | |
| --- | --- | --- |
| **Name** | **Company** | **Cat. Nr.** |
| Elispot Flex: Mouse IgG (ALP) | MABTECH | 3825-2A |
| Elispot Flex: Mouse IgM (ALP) | MABTECH | 3885-2A |
| Fixable viability dye | BioLegend | 423101 |
| Fix and Perm Cell Permeabilization Kit | ThermoFischer Scientific | GAS004 |
| Pan B cell isolation Kit II, Mouse | Miltenyi biotec | 130-104-443 |
| CD19 MicroBeads, mouse | Miltenyi Biotech |  |
| Opal 6-Plex Detectiom Kit | Akoya Bioscience | NEL811001KT |
| RNAscope Multiplex Flurescent Reagent Kit V2 | Biotechne | UM323100 |
| Phagocytosis Assay Kit (Red Zymosan) | Abcam | ab234054 |
| ReliaPrep™ RNA Cell Miniprep System Technical Manual | Promega | Z6012 |
| RevertAid First Strand cDNA Synthesis Kit | ThermoFischer Scientific | K1622 |
| SYBR Green q-RTPCR Master Mix | NEB | M3003E |
| CD8a+ T cell isolation kit | Miltenyi Biotec | 130-104-075 |
| Mouse T-activator CD3/CD28 Dynabeads | Thermo Fisher Scientific | 11456D |

**Supplementary Table 3**

| List of primers used for the VDJ recombination analysis | | |
| --- | --- | --- |
| ***Allele*** | **Primer seq** | **Direction** |
| SRP20 | GATTACCGCAGGAGGAGT | FWD |
|  | AGAACGGATGATTGGGAA | REV |
| mVH 7183 | GCG AAG CTT GTG GAG TCT GGG GGA GGC TTA | FWD |
| mVH J-558 | GCG AAG CTT ARG CCT RCT TCA GTG AAG | FWD |
| mJH_3 | AGG CTC TGA CCC TAG ACA G | REV |
| Vkdeg | GGCTGC TTC AGT GGC AGT GGR TCW GGR AC | FWD |
| Jk2AR | TTA GAC TTA GTG AAC AAG AGT TGA GAA | REV |
| Jk5BR | CGT CAA CTG ATA ATG AGC CCT CTC | REV |

**Supplementary Table 4**

| **List of primers used for the qRT-PCR** | | | |
| --- | --- | --- | --- |
| **Target** | **Assay Name** | **Gene Globe ID** | **Manufacturer** |
| *Gapdh* | Mm_Gapdh_3_SG | QT01658692 | QIAGEN |
| *CD79a* | Mm_Cd79a_1_SG | QT00097111 | QIAGEN |
| *CD79b* | Mm_Cd79b_1_SG | QT00243663 | QIAGEN |
| *Pax5* | Mm_Pax5_1_SG | QT00174398 | QIAGEN |
| *CD19* | Mm_Cd19_1_SG | QT00108801 | QIAGEN |
| *Itgam* | Mm_Itgam_1_SG | QT00156471 | QIAGEN |
| *Csf1r* | Mm_Csf1r_1_SG | QT01055810 | QIAGEN |
| *CD68* | Mm_Cd68_1_SG | QT00254051 | QIAGEN |
| *Emr1* | Mm_Emr1_1_SG | QT00099617 | QIAGEN |
| *Rag2* | Mm_Rag2_1_SG | QT00253414 | QIAGEN |
| *Foxo1* | Mm_Foxo1_1_SG | QT00116186 | QIAGEN |
| *Ebf1* | Mm_Ebf1_1_SG | QT00142884 | QIAGEN |
