# Supplementary material for: Pancreatic cancer induces B cell lineage plasticity via Pax5 inhibition to sustain immunosuppression
Source: Cell Death Discov. 2026 Jun 2;12:265. doi: 10.1038/s41420-026-03174-z (PMC13230850; doi:10.1038/s41420-026-03174-z)

Original Data Files

Original image of the IgM Elispot Assay showed in Figure 1J

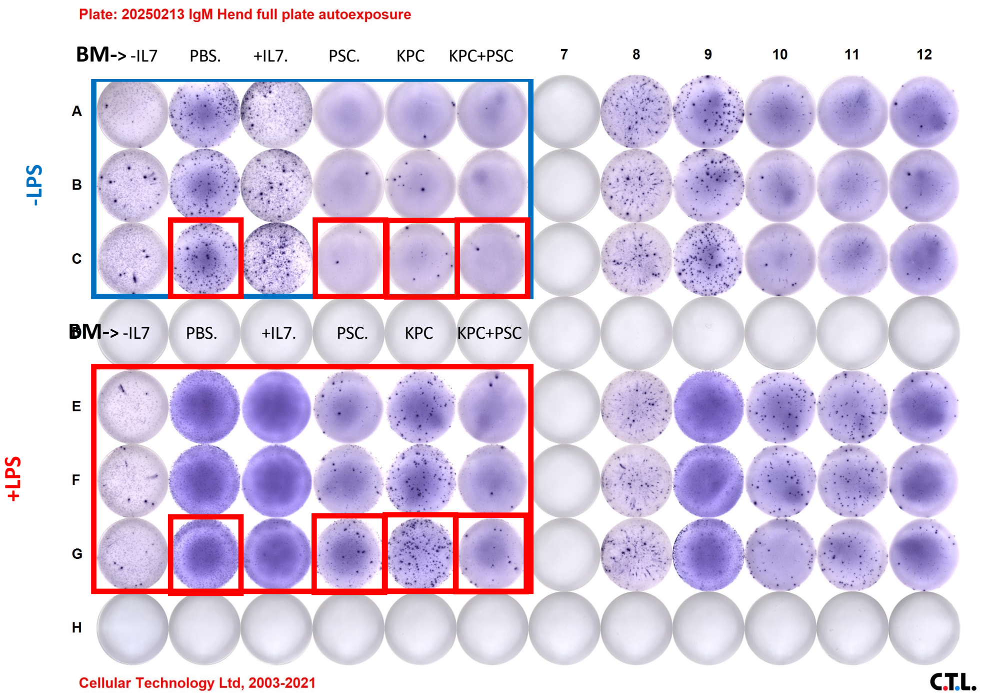

Used in the figure

Original uncropped image of the plate readout of IgM ELISpot responses to different culture conditions for 3 d. B cells cultured in medium supplemented with 5% FBS served as a control. B cells were stimulated with 2.5 µg/ml LPS (+LPS) or left without stimulation (-LPS).

Original image of the IgG Elispot Assay showed in Figure 1K

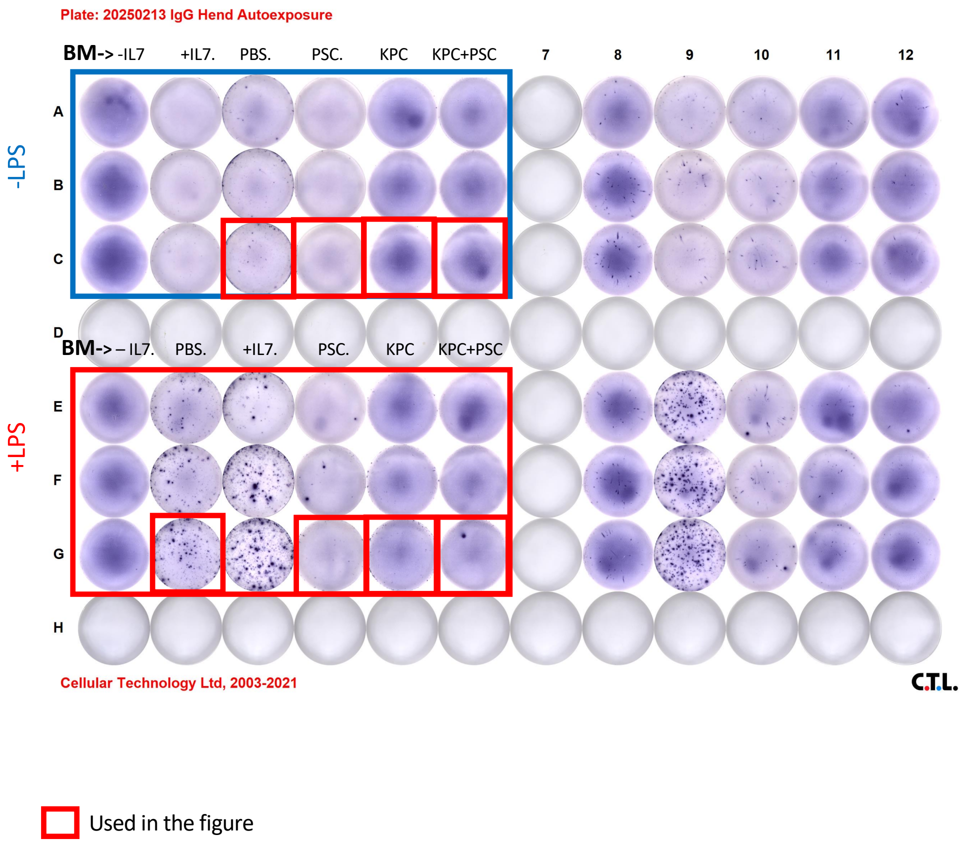

Original uncropped image of the plate readout of IgG ELISpot responses to different culture conditions for 3 d. B cells cultured in medium supplemented with 5% FBS served as a control. B cells were stimulated with 2.5 µg/ml LPS (+LPS) or left without stimulation (-LPS).

Original images of the *IgK VJ* recombination analysis showed in Figure 2J

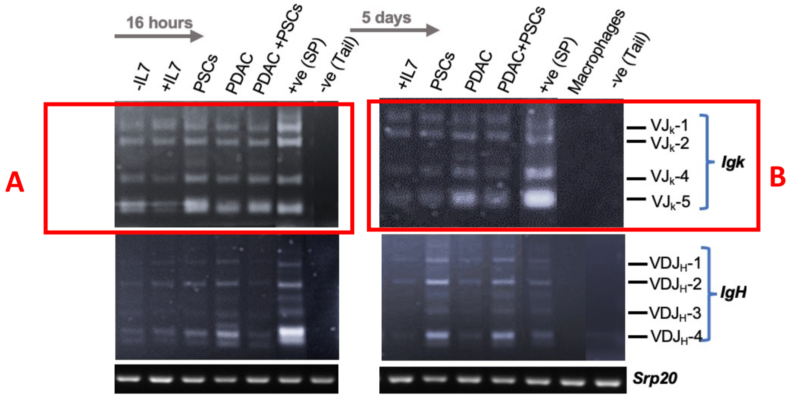

A) VJ recombination of IgK Light chain (16 h post co-culture)

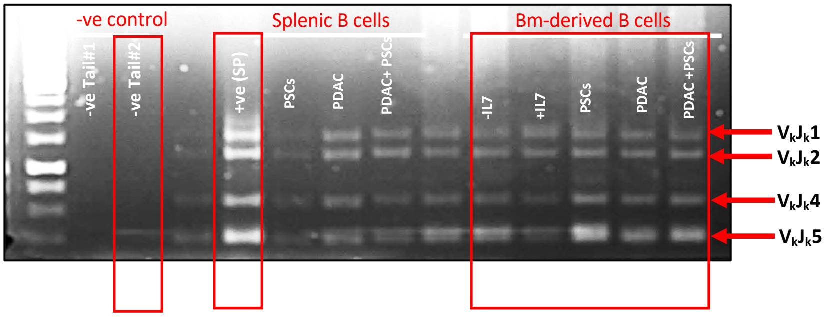

B) VJ recombination of IgK Light chain (5 d post co-culture)

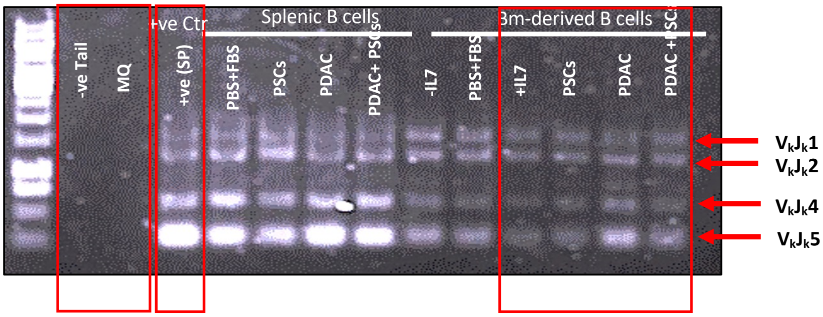

Used in the figure

Original uncropped images semi-quantitative PCR of the *Igk* rearrangements of the V_κ gene segments to $J_\kappa 1$, $J_\kappa 2$, $J_\kappa 4$, *and* $J_\kappa 5$ gene segments. Genomic DNA (gDNA) was isolated from B cells cultured under PDAC conditions as specified after **A)**16 h or **B)** 5 d of culture. GDNA isolated from splenic B cells was utilized as a +ve control for *V(D)J* recombination assay, while gDNA isolated from the mouse tail served as a technical –ve control for the recombination analysis. For comparison, a culture of conventional macrophages was generated concurrently with the B cell treatment. B cells cultured either in medium supplemented with IL7 (+IL7) or without IL7 (-IL7) were used as +ve and -ve controls for survival, respectively.

Original images of the *IgH VDJ* recombination analysis showed in Figure 2J

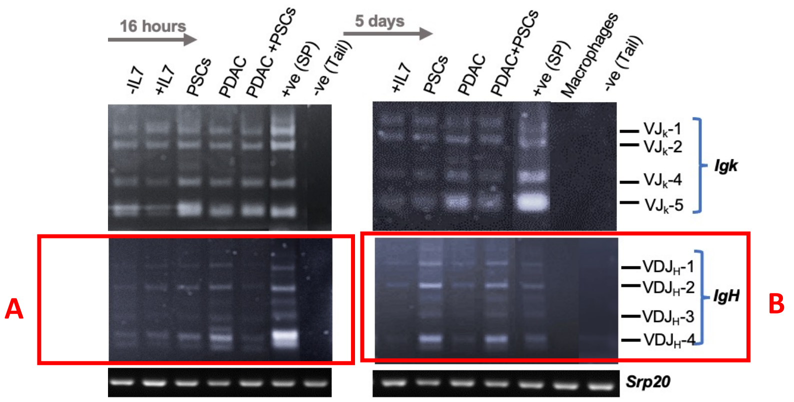

A) *VDJ* recombination of the Heavy chain *IgH* (16 h post co-culture)

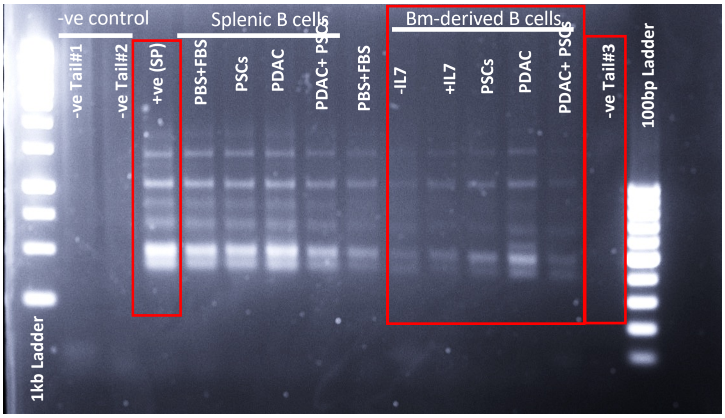

B) *VDJ* recombination of the Heavy chain *IgH* (5 d post co-culture)

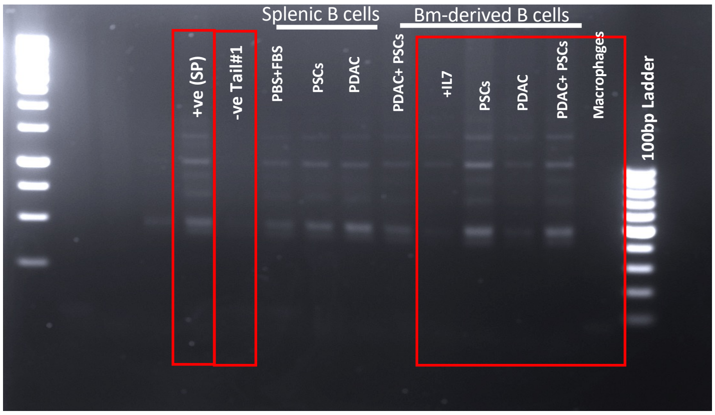

Original uncropped images of the semi-quantitative PCR of the *IgH* rearrangements of the proximal VD_h gene segments to J_H1, J_H2, J_H3, *and* J_H4 gene segments. Genomic DNA (gDNA) was isolated from B cells cultured under PDAC conditions as specified after **A)** 16 h or **B)** 5 d of culture. GDNA isolated from splenic B cells was utilized as a +ve control for *V(D)J* recombination assay, while gDNA isolated from the mouse tail served as a technical –ve control for the recombination analysis. For comparison, a culture of conventional macrophages was generated concurrently with the B cell treatment. B cells cultured either in medium supplemented with IL7 (+IL7) or without IL7 (-IL7) were used as +ve and -ve controls for survival, respectively.

Original images of the loading control *(Srp20)* showed in Figure 2J

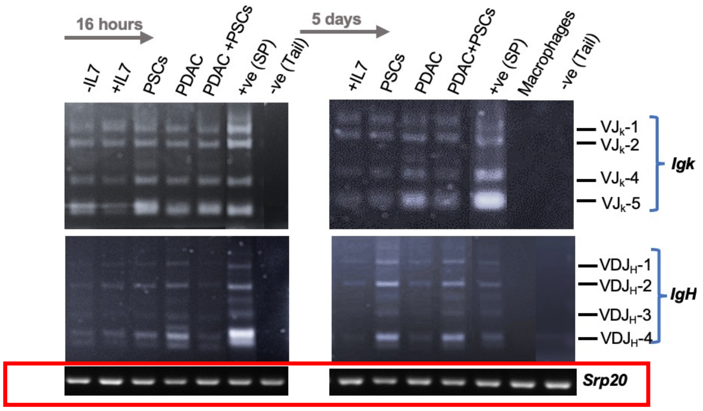

Loading Control *Srp20*

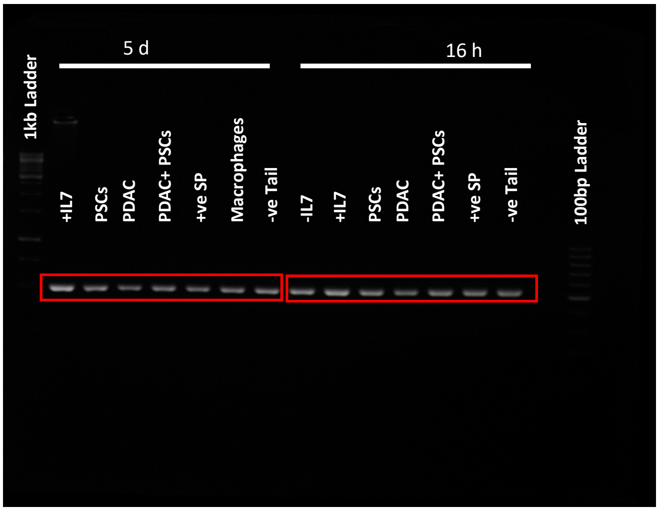

Original uncropped image of the semi-quantitative PCR Semi-quantitative PCR of the loading control *SRP20* for the PCR showed in figure 2J.

Original images of the *IgK VJ* recombination analysis showed in Figure 5K

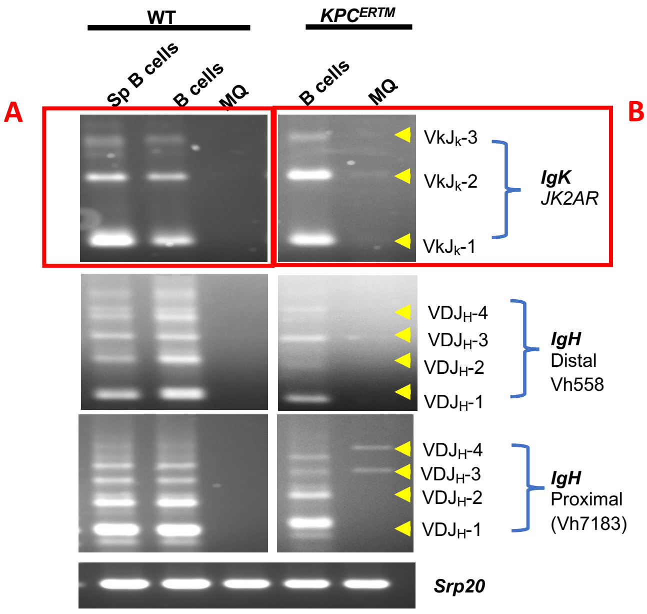

A) VJ recombination of *IgK* Light chain *Ig* in B cells and macrophages isolated from the bone marrow of WT mouse

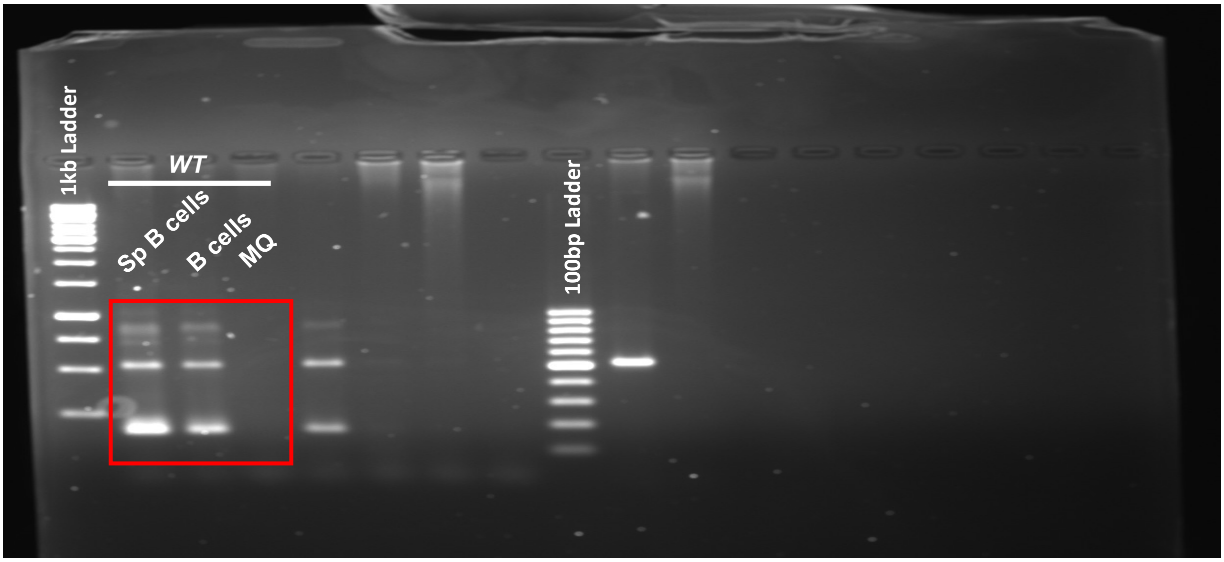

B) VJ recombination of *IgK* Light chain *Ig* in B cells and macrophages isolated from the bone marrow of *KPC* ^{ERTM} mice

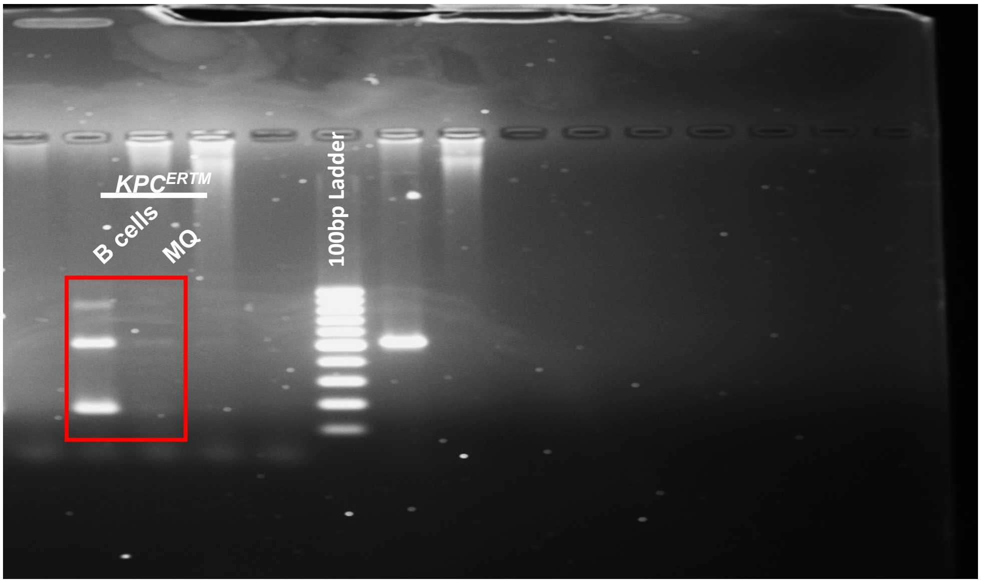

Original images of the *IgK VJ* recombination analysis showed in Figure 5K

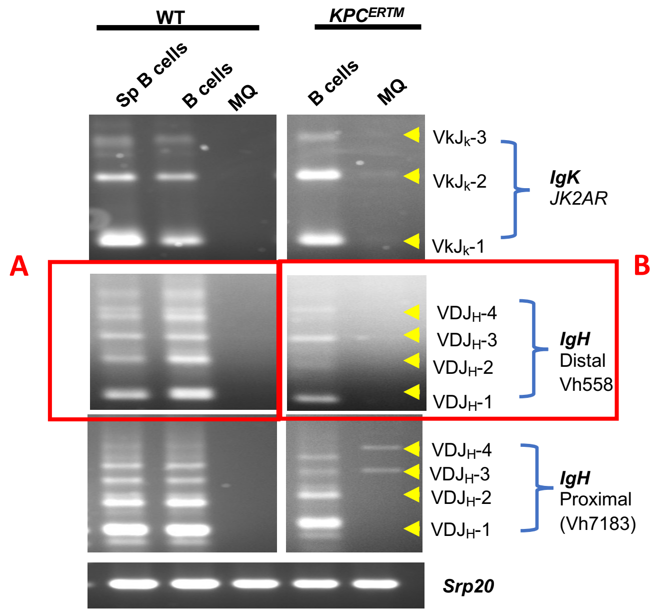

A) VJ recombination of *IgK* Light chain *Ig* in B cells and macrophages isolated from the bone marrow of WT mouse

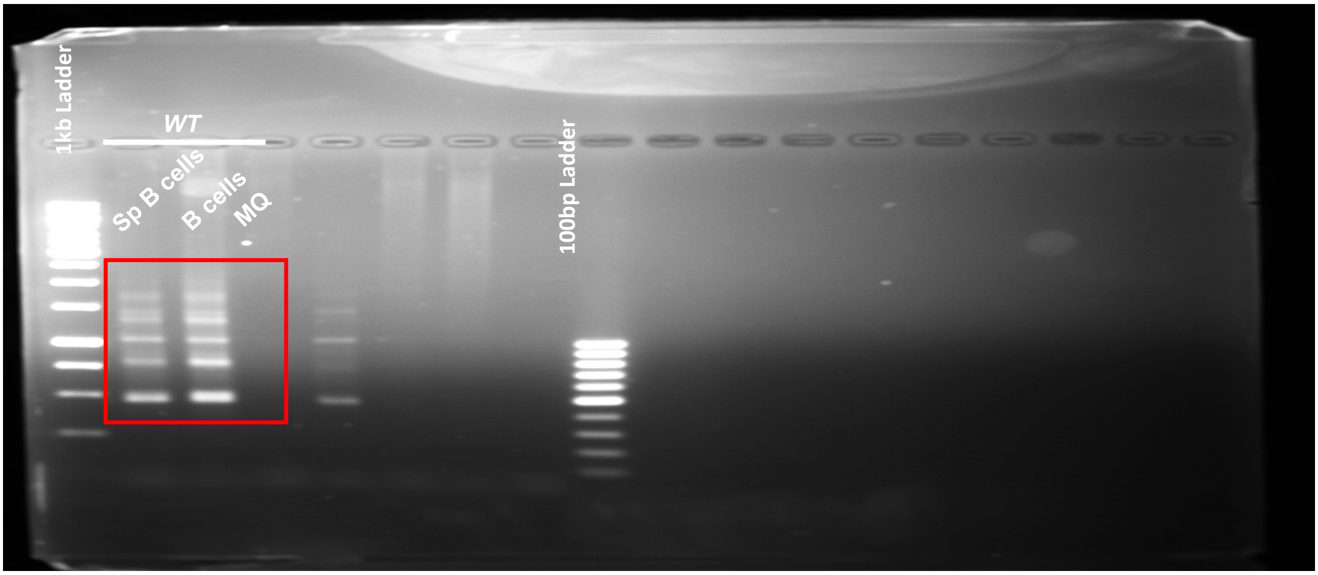

B) VJ recombination of *IgK* Light chain *Ig* in B cells and macrophages isolated from the bone marrow of *KPC* ^{ERTM} mice

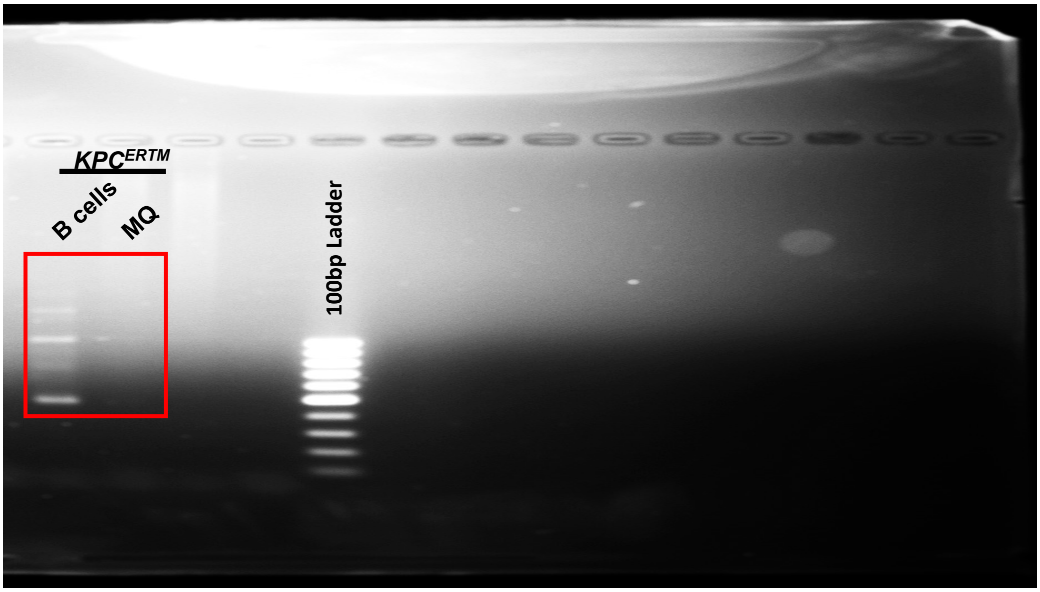

Original images of the *IgK VJ* recombination analysis showed in Figure 5K

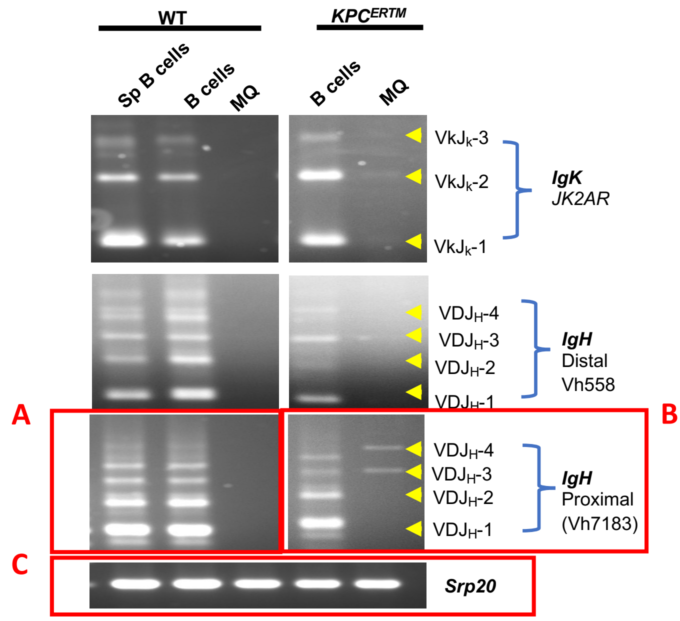

A) VDJ recombination of *IgH* in B cells and macrophages isolated from the bone marrow of *WT* and *KPC*^{ERTM} mice

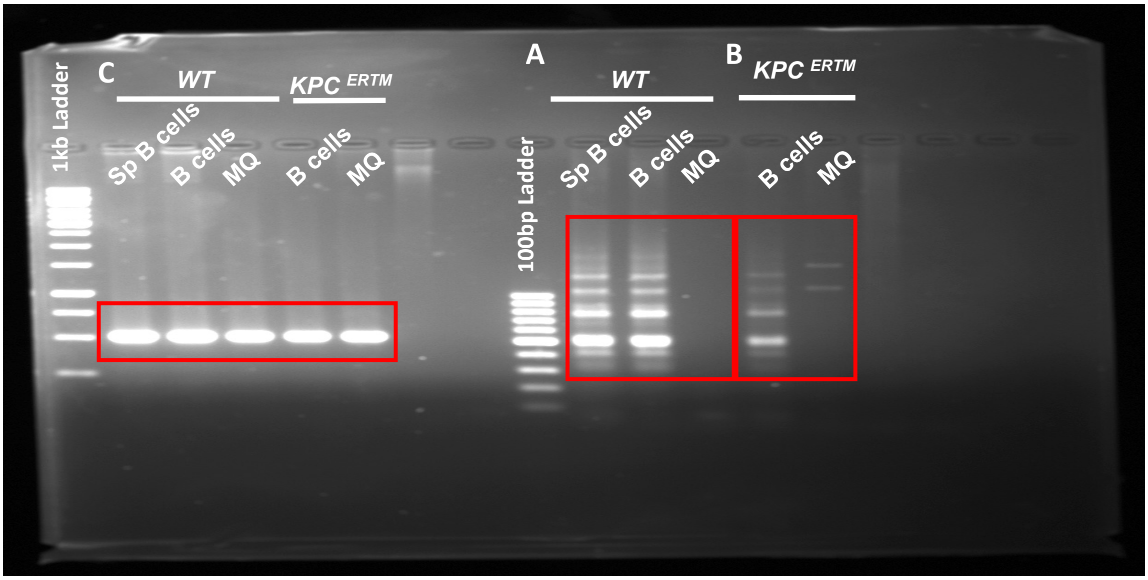

Supplement: Supplementary file 3 — Uncropped Figures [file 41420_2026_3174_MOESM3_ESM.pdf]
